# Beyond beta rhythms: subthalamic aperiodic broadband power scales with Parkinson's disease severity–a cross-sectional multicentre study

**DOI:** 10.1016/j.ebiom.2025.105988

**Published:** 2025-10-29

**Authors:** Moritz Gerster, Gunnar Waterstraat, Thomas S. Binns, Natasha Darcy, Christoph Wiest, Richard M. Köhler, Jojo Vanhoecke, Timothy O. West, Matthias Sure, Dmitrii Todorov, Lukasz Radzinski, Jeroen Habets, Johannes L. Busch, Lucia K. Feldmann, Patricia Krause, Katharina Faust, Gerd-Helge Schneider, Keyoumars Ashkan, Erlick Pereira, Harith Akram, Ludvic Zrinzo, Benjamin Blankertz, Arno Villringer, Huiling Tan, Jan Hirschmann, Andrea A. Kühn, Esther Florin, Alfons Schnitzler, Ashwini Oswal, Vladimir Litvak, Wolf-Julian Neumann, Gabriel Curio, Vadim Nikulin

**Affiliations:** a Department of Neurology, Max Planck Institute for Human Cognitive and Brain Sciences, Leipzig, Germany; b Bernstein Center for Computational Neuroscience, Berlin, Germany; c Neurophysics Group, Department of Neurology, Charité – Universitätsmedizin Berlin, Corporate Member of Freie Universität Berlin and Humboldt-Universität zu Berlin, Hindenburgdamm 30, 12203, Berlin, Germany; d Movement Disorder and Neuromodulation Unit, Department of Neurology, Charité – Universitätsmedizin Berlin, Corporate Member of Freie Universität Berlin and Humboldt Universität zu Berlin, Chariteplatz 1, 10117, Berlin, Germany; e Einstein Center for Neurosciences Berlin, Charité – Universitätsmedizin Berlin, Corporate Member of Freie Universität Berlin and Humboldt Universität zu Berlin, Chariteplatz 1, 10117, Berlin, Germany; f MRC Brain Networks Dynamics Unit, Nuffield Department of Clinical Neurosciences, University of Oxford, Oxford, UK; g Institute of Clinical Neuroscience and Medical Psychology, Medical Faculty, Heinrich-Heine University Düsseldorf, Düsseldorf, Germany; h Department of Neurosurgery, Charité – Universitätsmedizin Berlin, Corporate Member of Freie Universität Berlin and Humboldt-Universität zu Berlin, Berlin, Germany; i Department of Neurosurgery, King's College Hospital NHS Foundation Trust, London, SE5 9RS, UK; j City St George's, University of London & St George's University Hospitals NHS Foundation Trust, London, SW17 0QT, UK; k Unit of Functional Neurosurgery, Department of Clinical and Movement Neurosciences, UCL Queen Square Institute of Neurology, London, UK; l Neurotechnology Group, Technische Universität Berlin, Berlin, Germany; m Department of Imaging Neuroscience, UCL Queen Square Institute of Neurology, University College London, London, WC1N 3AR, UK; n The Biomedical Imaging Laboratory INSERM U1146, CNRS UMR 7371, Sorbonne University, 15 Rue de l’Ecole de Médecine, 75006, Paris, France; o Department of Bioengineering, Sir Michael Uren Hub, Imperial College London, London, W12 0BZ, UK

**Keywords:** Basal ganglia, Movement disorders, Neurodegenerative disorders, Spectral parameterization, Reproducibility, 1/f activity

## Abstract

**Background:**

Parkinson's disease is linked to increased beta rhythms (13–30 Hz) in the subthalamic nucleus, which correlate with motor symptoms. However, findings across studies are inconsistent. Furthermore, the contribution of other frequencies to symptom severity remains underexplored.

**Methods:**

We analysed subthalamic local field potentials from 119 patients with Parkinson's disease (31 female; mean age 60 ± 9 years) across five independent datasets. Power spectra were parametrised and studied in relation to Levodopa administration and the severity of motor symptoms.

**Findings:**

Our findings suggest that small sample sizes contributed to the variable correlations between beta power and motor symptoms reported in previous studies. Here, we demonstrate that more than 100 patients are required for stable replication. Aperiodic offset and low gamma (30–45 Hz) oscillations were negatively correlated with motor deficits ($r_{\text{Offset}}=-0.32$, $p=4e^{-4}$; $r_{L\gamma }=-0.21$, p=0.021), whereas low beta oscillations were positively correlated ($r_{L\beta }=0.24$, p=0.010). Combining offset, low beta, and low gamma power ($r_{\text{Lin}.\;\text{reg}.\;\left(\text{Offset},L\beta ,L\gamma \right)}=0.47$, p=1e−4) explained significantly more variance in symptom severity than low beta alone (*J*-test: $p=2e^{-5}$). Interhemispheric within-patient analyses showed that, unlike beta oscillations, aperiodic broadband power (2–60 Hz)–likely reflecting spiking activity–was increased in the more affected hemisphere (Levodopa off-state: p=0.015; on-state: p=0.005).

**Interpretation:**

Spectral features beyond conventional beta rhythms are critical to understanding Parkinson's pathophysiology. Aperiodic broadband power shows potential as a new biomarker for adaptive deep brain stimulation, providing important insights into the relationship between subthalamic hyperactivity and motor symptoms in Parkinson's disease.

**Funding:**

This work was supported by 10.13039/501100001659Deutsche Forschungsgemeinschaft (German Research Foundation) Project ID 424778381 TRR 295 “ReTune”. H.A. is supported by 10.13039/501100000272NIHR UCLH BRC. This work was supported by an 10.13039/501100000265MRC Clinician Scientist Fellowship (MR/W024810/1) held by A.O. W.-J.N. received funding from the 10.13039/501100000780European Union (ERC, ReinforceBG, project 101077060). E.F. received funding from the Volkswagen foundation (Lichtenberg program 89387). G.W. and L.R. received funding from 10.13039/501100001659Deutsche Forschungsgemeinschaft Project ID 511192033.

**Research in context**

**Evidence before this study**

Parkinson's disease can be treated with deep brain stimulation of the subthalamic nucleus, which also enables direct recordings of brain activity. Many studies investigated whether the power of beta rhythms (13–30 Hz) relates to the severity of motor symptoms. These studies varied widely in sample sizes (7–103 patients, median 13), the frequency ranges defined as “beta,” and statistical outcomes (17 significant vs. 22 non-significant correlations). These inconsistencies motivated our large-scale, standardised analysis.

**Added value of this study**

Integrating five datasets comprising 119 patients—far exceeding the typical sample sizes in prior studies—we demonstrate that inconsistencies in beta power vs. symptom correlations primarily stem from underpowered studies. By disentangling rhythmic from non-rhythmic brain activity, we enhanced symptom associations and improved physiological specificity. Moreover, we identified aperiodic broadband power as a marker that reflects symptom severity at the individual level, applicable across medication states. Total mid gamma power (45–60 Hz) also tracked symptom asymmetry.

**Implications of all the available evidence**

Our findings underscore the importance of large datasets and physiologically grounded, multiparametric spectral analysis for biomarker discovery. Aperiodic broadband power is a promising spiking-related marker for invasive electrophysiology. Total mid gamma power, as a real-time extractable proxy of aperiodic broadband power, may enable dynamic symptom tracking relevant for adaptive deep brain stimulation in Parkinson's disease.

## Introduction

Parkinson's disease (PD) is characterised by progressive motor impairments due to basal ganglia dysfunction. Within the basal ganglia, abnormal subthalamic nucleus (STN) activity plays a central role, exhibiting two major abnormalities in PD: excessive beta (13–30 Hz) rhythms and increased neuronal spiking activity.^1^^,^^2^ Deep brain stimulation (DBS) of the STN alleviates motor symptoms and enables local field potential (LFP) recordings.^3^^,^^4^ Since LFPs primarily capture oscillatory activity rather than spiking, research in humans has been skewed toward studying beta oscillations.

This research focus on beta has led to numerous reports linking subthalamic beta activity with motor impairment in PD.^5^ These reports inspired beta-based adaptive DBS (aDBS)—a closed-loop stimulation approach that uses beta power as an electrophysiological biomarker to adapt DBS dynamically.^6^ Early trials suggest aDBS may outperform continuous DBS,^7^ but a deeper characterisation of the beta–symptom correlation may refine its clinical application. Furthermore, current aDBS implementations assume that beta power most reliably reflects motor dysfunction—a premise that remains uncertain.

While the beta–symptom correlation has been extensively studied (Supplementary Table S2), its *robustness* is unclear due to methodological variability; its *replicability* awaits testing in large, diverse cohorts; and its *strength* varies considerably across studies.^8^, ^9^, ^10^, ^11^, ^12^ Furthermore, most studies use *across*-patient correlations, though aDBS biomarkers must track symptoms *within* individuals. Finally, beta–symptom correlations are predominantly studied in the Levodopa *off*-state, whereas most DBS patients remain *on* medication.

**Fig. 1:**
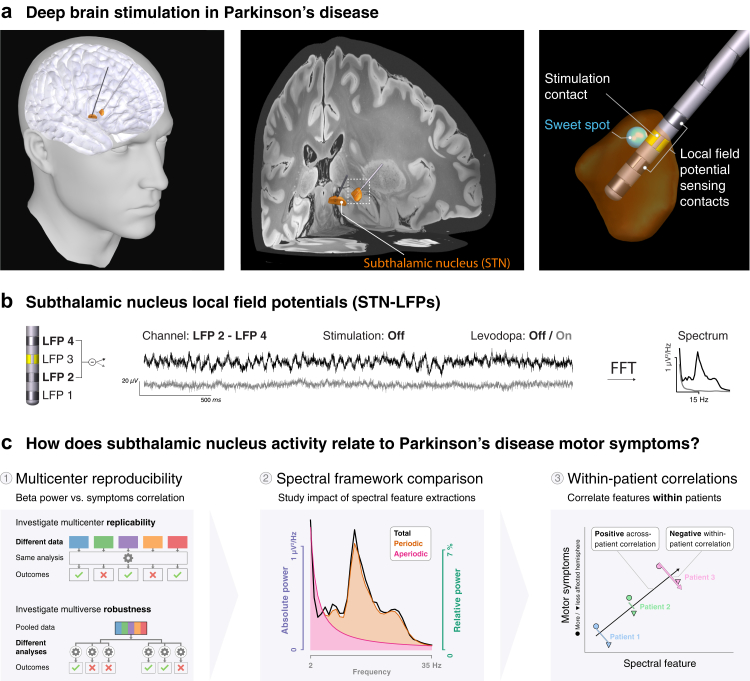
**Investigating the relationship between subthalamic nucleus activity and Parkinson's disease symptoms.** (**a**) Illustration of subthalamic nucleus (STN) deep brain stimulation (DBS) at three increasing anatomic scales. The 3D visualisations show a typical positioning of the DBS leads relative to the brain. The DBS contact closest to the stimulation sweet spot^30^ (blue sphere) is the estimated stimulation contact (yellow). (**b**) Left: The two contacts adjacent to the stimulation contact are referenced in a bipolar montage and used for analysis (DBS leads with directional contacts are averaged so all leads have four levels of contacts). Middle: Three seconds of raw bipolar local field potential (LFP) time-series traces from one exemplary patient with and without Levodopa administration. Right: Spectrum of the entire recording. The patient shows characteristic 14 Hz low beta oscillations off Levodopa (black), which disappear after Levodopa administration (grey). (**c**) Part 1: Multicentre reproducibility—the reproducibility of the correlation between beta power vs. motor symptoms is assessed in terms of replicability across datasets and robustness to different analyses. Part 2: Spectral framework comparison—different methods to extract spectral features, such as absolute total, relative total, and absolute periodic beta power, are evaluated for their motor symptom correlation. Part 3: Within-patient correlations—the relationships between spectral features and motor symptoms are tested for within-patient predictability. While within-patient correlations are imperative for successful aDBS, these might not be truthfully reflected in across-patient correlations.

To address these challenges, we conducted a multicentre STN-LFP analysis, integrating five independent datasets (Fig. 1) to create a large and heterogeneous cohort of 119 patients with PD. In Part 1, we extensively characterise the beta–symptom correlation. Part 2 compares three spectral analysis frameworks to determine which best reflects neural dynamics. In Part 3, we leverage PD's asymmetric nature and compare STN activity between more vs. less affected hemispheres, providing additional insights into how spectral features correlate with symptom lateralisation at the individual patient level. We identify spectral features that correlate *within* patients and *across* medication states—two requirements for aDBS biomarkers.

We find that aperiodic broadband power meets both requirements, offering a promising target for aDBS. It is strongly elevated in the more affected hemisphere and has been reported to correlate with spiking activity^13^—the second major abnormality in PD, which, however, remained understudied relative to beta synchronisation.

## Methods

### Study design

**Fig. 2:**
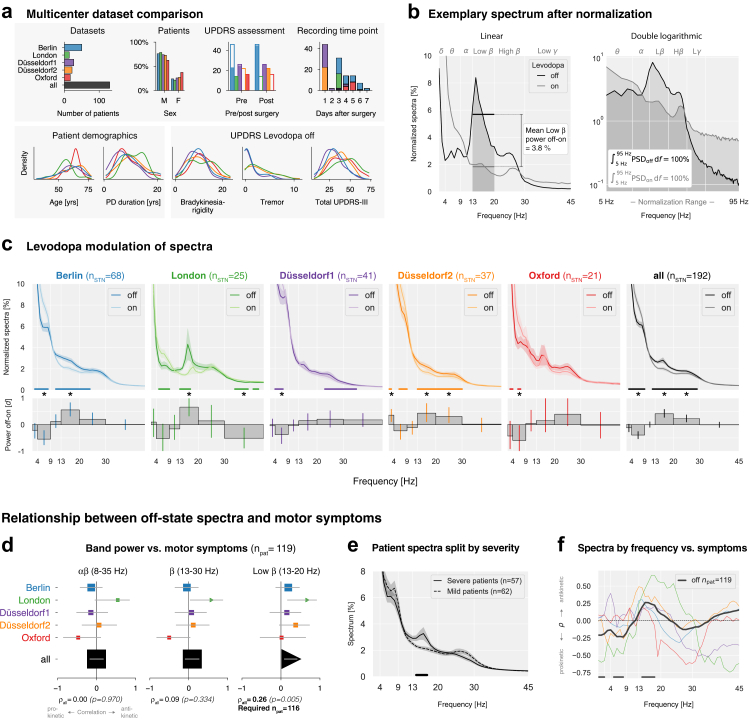
**Multicentre analysis of STN-LFP recordings in Parkinson's disease patients.** (**a**) Dataset and patient characteristics. Top row: Number of patients per dataset; sex distribution; time point of UPDRS-III assessment. Y-axis: number of patients, filled bars: proportion of evaluated patients; time point of recording. Bottom row: Kernel density estimates of age, disease duration, and UPDRS-III subscores, showing comparable demographic and clinical characteristics across datasets. Additional details on DBS lead manufacturers, localisations, and symptoms in the on-state are in Supplementary Fig. S1. (**b**) Exemplary STN-LFP spectrum from a single patient (Berlin) in the Levodopa off and on states, normalised to the 5–95 Hz frequency range. White vertical grid lines indicate canonical frequency band borders. Left: Total relative band power was calculated as the average power within each canonical frequency band. Right: Normalisation equalises the area under the spectral curve to 100% between 5 and 95 Hz. (**c**) Levodopa modulation of STN spectral power across datasets. Upper: Averaged spectra by dataset and Levodopa condition, shading shows standard error. Horizontal colored lines indicate frequency ranges with significant power differences. Below: Effect sizes (Cohen's d) for Levodopa-induced spectral changes. Vertical colored lines indicate 99th-percentile confidence intervals; asterisks note Bonferroni-corrected significant effect sizes (*p* < 0.008). Complete statistics in Table 2. Absolute spectra results are in Supplementary Fig. S1c. (**d**) Correlations between average band power vs. motor symptoms. X-axis: Spearman's correlation coefficient ρ, y-axis: datasets, horizontal lines: 95th percentile confidence intervals, symbol sizes represent the dataset sample sizes. Triangles indicate significant correlations (*p* < 0.05); squares indicate non-significant findings. Pooled correlation coefficients and p-values are shown at the bottom. “Required n_pat_” indicates sample size estimations (80% power requirement) for the observed correlation coefficients. (**e**) Patient spectra split by median UPDRS-III score after averaging their hemispheres. The horizontal line shows a 14–17 Hz cluster of significant difference. (**f**) Frequency-wise correlation between relative spectral power and motor symptoms. Horizontal lines indicate frequencies with uncorrected p-values < 0.05 for the pooled data.

This is a cross-sectional, observational, multicentre analysis of resting-state STN-LFP recordings from patients with PD. We analysed five independent datasets: Berlin,^14^, ^15^, ^16^ London,^17^ Düsseldorf 1,^18^, ^19^, ^20^, ^21^, ^22^ Düsseldorf 2,^23^, ^24^, ^25^ and Oxford.^26^, ^27^, ^28^ The patient demography, disease characteristics, and acquisition details are presented in Table 1, Fig. 2a, Supplementary Fig. S1, and Supplementary Table S1. Participant sex was obtained from clinical records (biological sex; male/female). No self-reported gender information was available. Sex data were used for demographic description; exploratory analyses showed no significant influence of sex on the reported results.

**Table 1:** Patient demographics (mean ± SD).

Characteristics	All (*n* = 130)	Female (*n* = 34)	Male (*n* = 95)
Age [years]	60.0 ± 8.5	63.0 ± 6.7	59.1 ± 8.8
Disease duration [years]	9.6 ± 4.6	9.1 ± 4.5	9.8 ± 4.6
UPDRS-III (off)	34.9 ± 12.9	33.3 ± 12.9	35.3 ± 12.9
UPDRS-III (on)	19.0 ± 9.2	21.0 ± 8.7	18.5 ± 9.2

Sample sizes differ across analyses throughout the paper depending on the availability of UPDRS scores, MNI coordinates, and medication condition. Sex information was unavailable for one patient. UPDRS-III: Unified Parkinson's Disease Rating Scale Part III (motor symptom severity).

### Surgery

All patients were diagnosed with idiopathic PD of primary bradykinetic-rigid motor phenotype and underwent bilateral DBS lead implantation. Recruitment sites were: Berlin (*n* = 50, Charité – Universitätsmedizin Berlin), London (*n* = 14, National Hospital of Neurology and Neurosurgery), Düsseldorf 1 (*n* = 27) and Düsseldorf 2 (*n* = 22, University Hospital Düsseldorf), and Oxford (*n* = 17, St. George's University Hospital NHS Foundation Trust and King's College Hospital NHS Foundation Trust). Intraoperative microelectrode recordings were performed for the Berlin dataset (*n* = 2), Düsseldorf 1 (mean ± SD: 3.4 ±1.2), and Düsseldorf 2 (up to 5). Final DBS lead positions were reconstructed using Lead-DBS^29^ for Berlin, London, and Düsseldorf 1 (Supplementary Fig. S1b).

### Levodopa administration

For the off-state evaluation, patients were withdrawn from all dopaminergic medication for ≥12 h. For the on-state, patients received Levodopa ≥30 min before assessment, and movement disorder neurologists confirmed a clear motor on-state. Patients received either their usual dose of Levodopa (Berlin, London, Oxford) or 1.5 times their usual dose (Düsseldorf 1, Düsseldorf 2).

### Symptom evaluation

Movement disorder neurologists evaluated motor symptoms using the Unified Parkinson's Disease Rating Scale Part 3 (UPDRS-III) with and without Levodopa medication. Assessments were conducted pre-operatively for London and Oxford, post-operatively for Düsseldorf 2, and both pre- and post-operatively for Berlin and Düsseldorf 1 (Fig. 2a). The total UPDRS-III score was used as a patient-level measure of motor impairment. Hemisphere-specific bradykinesia-rigidity and tremor subscores were calculated by summing contralateral UPDRS items 22–26 and 20–21, respectively.

### Recordings

Recordings were performed 1–7 days after surgery while electrodes remained externalised (Fig. 2a). On- and off-state recordings took place on the same day for Düsseldorf 1, Düsseldorf 2, and Oxford, and on different days for Berlin (off: day 4.3±1.3, on: 3.8±1.5) and London (day 2 or 3 counterbalanced across patients). For the resting-state recordings, patients were instructed to rest with their eyes open for ≥3 min. Amplifiers, DBS lead models, recording sample rates, hardware filters, and recording references differed across datasets (Supplementary Fig. S1a, Supplementary Table S1). However, multicentre LFPs were harmonised using a standardised processing pipeline that involved conversion to microvolts, downsampling, filtering within the original hardware ranges, and bipolar re-referencing.

### Ethics

All patients provided informed consent to participate in the research, and recordings were performed according to the standards set by the Declaration of Helsinki. The study protocol for Berlin was approved by the ethics committee at Charité Universitätsmedizin Berlin (EA2/129/17), for Düsseldorf 1 and 2 by the ethics committee of the medical faculty of Heinrich Heine University Düsseldorf (study no. 3209 and 5608R), for London by the joint ethics committee of the National Hospital of Neurology and Neurosurgery and the University College London Institute of Neurology (07/Q0512/10), and for Oxford by the Health Research Authority UK, the National Research Ethics Service local Research Ethics Committee (IRAS: 46576), and the South Central–Oxford C Research Ethics Committee (19/SC/0550).

### Signal processing

#### Preprocessing

All recordings were manually screened to reject bad segments and channels, then high-pass filtered at 1 Hz. Before downsampling to 2000 Hz, a low-pass filter was applied to prevent aliasing.

#### Channel selection

Directional DBS contacts were averaged at each level along the electrode shaft, and we estimated the most likely monopolar stimulation contact (either contact 2 or 3) based on proximity to the DBS sweet spot.^30^ When MNI coordinates were unknown, we estimated the stimulation contact based on the largest high beta power.^31^ For each STN, we select one bipolar LFP for further analysis. We chose the neighbouring electrodes adjacent to the estimated stimulation contact, either contacts 1–3 or 2–4, as suggested for aDBS sensing.^6^^,^^32^

#### Absolute spectra

Power spectra were computed using the Welch algorithm with a frequency resolution of 1 Hz (1-s Hamming windows) and 50% overlap between neighbouring windows. Line noise artefacts were linearly interpolated in the spectrum.

#### Relative spectra

Relative power spectra in percentage units were obtained by dividing the absolute spectra by their sum from 5 to 95 Hz and multiplying by 100% (Fig. 2b, right).^33^

#### Parameterized spectra

We applied *specparam*^34^ (formerly ‘FOOOF’) to separate periodic and aperiodic spectral components with the following parameters: fit range: 2–60 Hz, peak width limits: 2–12 Hz, maximum number of peaks: 4, minimum peak height: 0.1, peak threshold: 2, aperiodic mode: fixed. Fits with $R^{2}$ values above 0.85 were kept for further processing.^35^

#### Band power

Band power was obtained from the total or periodic spectra by selecting the average power in the canonical frequency bands delta (2–4 Hz), theta (4–9 Hz), alpha (9–13 Hz), low beta (13–20 Hz), high beta (20–30 Hz), low gamma (30–45 Hz), and mid gamma (45–60 Hz).

#### Aperiodic broadband power

Aperiodic broadband power can be calculated from the offset a and the 1/f exponent m by summing the fitted aperiodic power from $f_{\text{low}}=2\;\text{Hz}$ to $f_{\text{high}}=60\;Hz$:

$$\text{Aperiodic}\;\text{power}=a\cdot \left(f_{\text{high}}-f_{\text{low}}\right)\;-\;m\sum_{f=f_{\text{low}}}^{f_{\text{high}}}\;\log_{10}\left(f\right)$$

Please refer to the supplementary material for a Python implementation using *specparam*.

### STN-LFP simulations

STN-LFP power spectra were simulated by constructing a Fourier power spectrum following a preset $1/f^{m}$ power law. The corresponding phases of the Fourier spectrum are distributed uniformly randomly. To add oscillations, we add Gaussian-shaped peaks to the Fourier power spectrum with amplitudes A and a spectral extent given by centre frequencies $f_{\text{centre}}$ and variances $\sigma_{f}^{2}$. The corresponding time series, consisting of periodic oscillations and aperiodic activity, is then obtained by applying the inverse fast Fourier transform. The simulated time series have a duration of 180 s at a sampling rate of $f_{\text{sample}}=2400\;\text{Hz}$.

### Spatial localisation of oscillations

The spatial localisation of oscillatory activity was conducted following protocols described in Horn et al.^36^ and Darcy et al.,^31^ pooling datasets from Berlin, London, and Düsseldorf 1, where MNI coordinates were available. To maximise spatial resolution, we analysed adjacent bipolar channel pairs (1–2, 2–3, 3–4), and the site of maximum band power within each STN was identified. The power was mapped into MNI space, using the midpoint between bipolar recording coordinates, resulting in a 4D grid (3D spatial coordinates + power values) for each frequency band and levodopa condition. Interpolations between data points were performed using a scattered interpolant, and the resulting maps were smoothed with a Gaussian kernel (FWHM=0.7mm)—the Lead-DBS software integrated subcortical parcellations from the DISTAL atlas.^37^ Left hemisphere coordinates were flipped non-linearly to the right to increase data density and facilitate visualisation. Finally, power values were thresholded at their mean plus one standard deviation to highlight the regions of interest, as shown in the thresholded probability maps in Fig. 5.

**Fig. 3:**
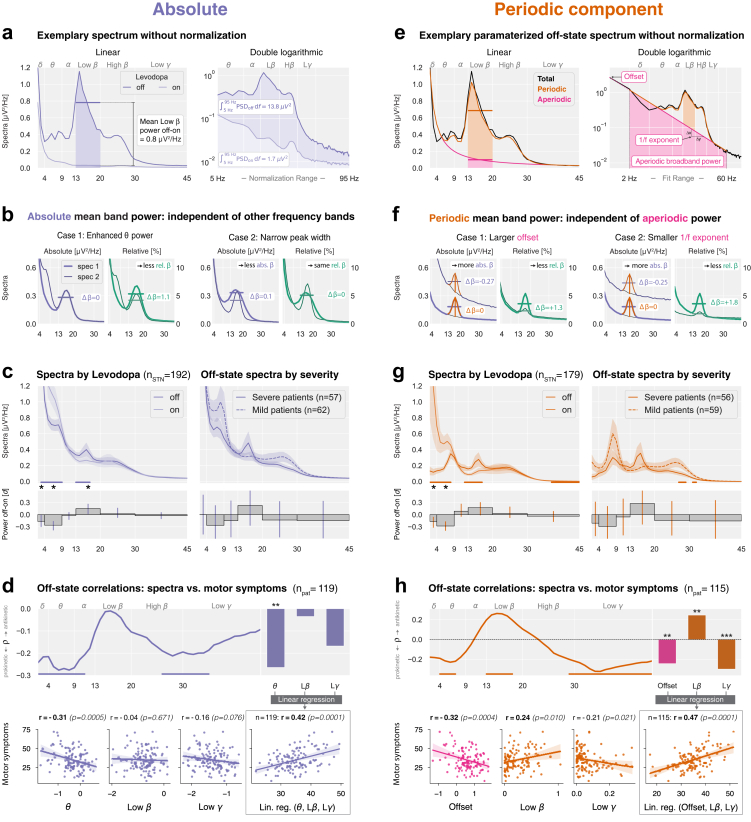
**Spectral components beyond beta are associated with symptom severity.** (**a**) Same spectrum as in Fig. 1b**,** but without normalisation. (**b**) Simulations highlight the advantages of investigating non-normalised spectra in absolute units over normalised spectra in relative units. (**c**) Left: Mean absolute spectra pooled across datasets. Bars show effect sizes, and horizontal lines indicate cluster statistics (paired statistics). Right: Levodopa off spectra for patients split by median UPDRS-III score (unpaired statistics). (**d**) Top left: Correlation between absolute power and motor symptoms across frequency bins. Top right: Correlation coefficients for band power in the theta, low beta, and low gamma ranges. Bottom left: Pearson correlations r for linear regression features. Bottom right: Linear regression model combining theta, low beta, and low gamma power. (**e**) Same spectrum as in (a) (off condition), but parameterised into periodic (orange) and aperiodic components (pink). The aperiodic component follows a 1/f power-law, modelled as $\text{Spectru}m_{\text{aperiodic}}=a+m\cdot \text{fre}q_{\log}$, where a represents the offset and m the exponent. (**f**) Simulations highlight the implications of investigating periodic power. **(g)** and **(h)**, Same as (c) and (d) for the periodic component and aperiodic offset.

**Fig. 4:**
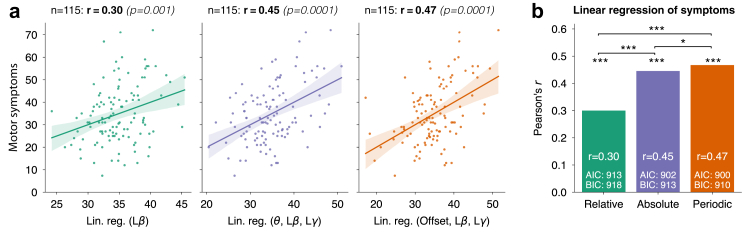
**Comparison of off-state linear regression models explaining motor symptoms.** (**a**) Linear regression models for relative total, absolute total, and absolute periodic power frameworks. The y-axis shows empirical motor symptoms (UPDRS-III scores), whereas the x-axis represents model predictions. Pearson's correlation coefficient r and statistical significance p are shown for each model. ‘Relative’ linear regression coefficients: $b_{0}=27.6$, $b_{L\beta }=22.3$; ‘Absolute’ coefficients: $b_{0}=26.0$, $b_{\theta }=-14.1$, $b_{L\beta }=12.9$, $b_{L\gamma }=-10.1$, ‘Periodic’ coefficients: $b_{0}=38.3$, $b_{\text{Offset}}=-6.7$, $b_{L\beta }=14.5$, $b_{L\gamma }=-45.1$. $b_{0}$ indicates the linear regression intercept. (**b**) The absolute and periodic three-parameter models outperform the one-parameter relative model, and the periodic model outperforms the absolute model.

**Fig. 5:**
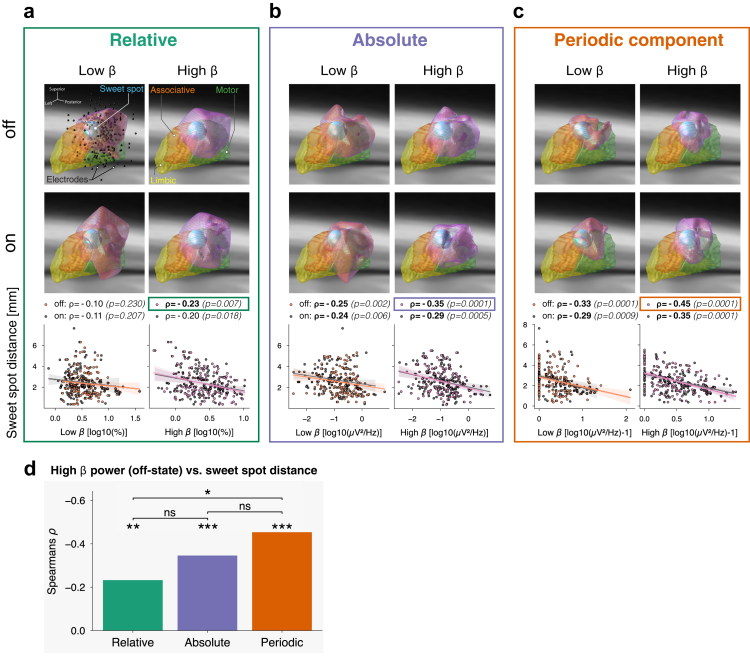
**Spatial localisation of beta oscillations in the subthalamic nucleus.** (**a**) Relative total power: Thresholded volumetric heatmaps show the spatial distribution of low beta (left) and high beta power (right) in the STN for Levodopa off (top) and on (middle) conditions. Electrode positions (black spheres) and the DBS sweet spot^30^ (blue sphere) are annotated. Bottom: Correlations between beta power and sweet spot distance, with each data point representing one STN. Significant correlations (Bonferroni-corrected for four comparisons, threshold p<0.013) are shown in bold. (**b**) Absolute total power: Same as (a) without normalisation. (**c**) Absolute periodic power: Same as (b) after removal of the aperiodic component. High beta power in the off condition shows substantial spatial concentration at the DBS sweet spot. Sample sizes (a)–(c) off: $n_{\text{STN}\;}=137$, on: $n_{\text{STN}\;}=132$. (**d**) Summary: Correlations between high beta power and sweet spot distance in the Levodopa off condition for all three spectral frameworks. Absolute periodic high beta power showed a significantly stronger correlation with DBS sweet spot distance than relative total high beta power.

**Fig. 6:**
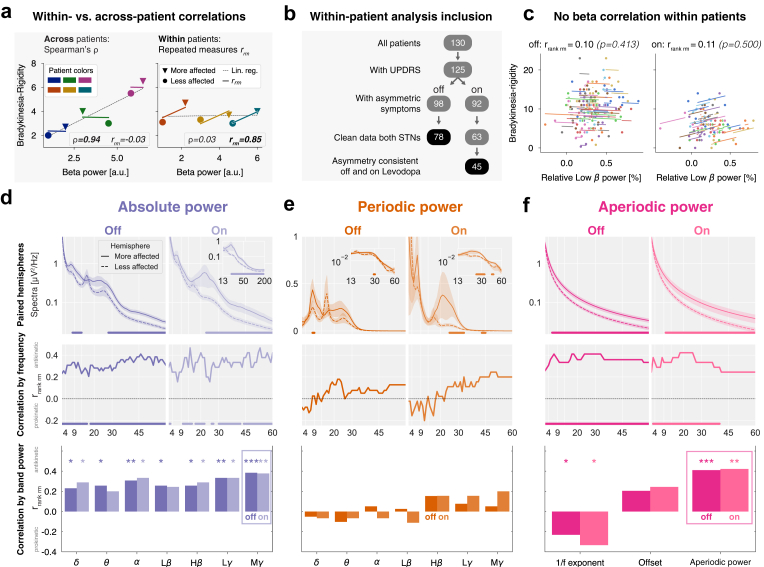
**Hemispheric aperiodic broadband power correlates with symptom severity within patients.** (**a**) Toy example. Across-patient (left) vs. within-patient (right) correlations between beta power and bradykinesia-rigidity subscores. Spearman's rank correlation ρ quantifies across-patient relationships, while repeated measures correlation $r_{\text{rm}}$ quantifies within-patient relationships. Each colour represents one patient, triangles and circles indicate more and less affected hemispheres. A strong across-patient correlation does not necessarily imply a strong within-patient correlation. (**b**) Inclusion criteria for within-patient analysis. Black background indicates the final number of patients for each Levodopa condition. (**c**) Repeated measures correlation scatter plot of relative low beta power (x-axis) and contralateral bradykinesia-rigidity subscore (y-axis). Each colour represents a different patient, and each patient is shown with two dots indicating both hemispheres. Colored slopes indicate linear (non-ranked) repeated measures correlation. The values on top provide the repeated-measures statistics after ranking the data. (**d**) Top: Absolute power within-patients paired cluster-based permutation tests for the more and less affected hemisphere in both Levodopa conditions. Middle: Repeated measures rank correlation for each frequency bin of the spectrum and the contralateral bradykinesia-rigidity subscore. Bottom: Repeated measures rank correlation for each band power. Significant correlations are marked with asterisks (‘∗’: p<0.05, ‘∗∗’: p<0.01, ‘∗∗∗’: p<0.001). (**e**) and (**f**) Same as (d) for periodic and aperiodic power. Aperiodic power indicates the sum of the aperiodic component from 2 to 60 Hz.

**Fig. 7:**
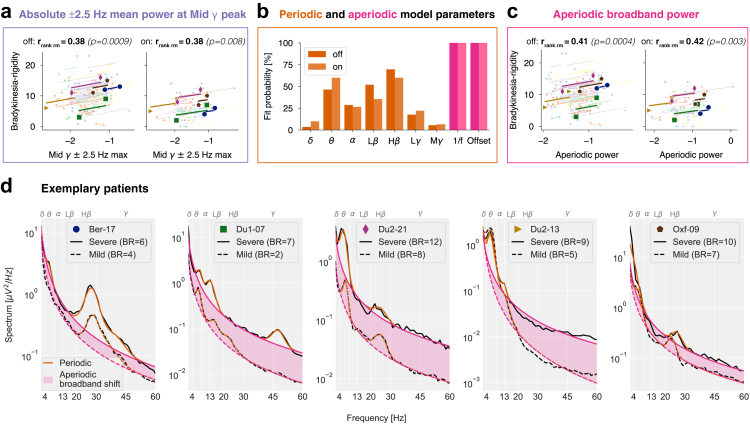
**Absolute total mid gamma power as a potential adaptive deep brain stimulation (aDBS) biomarker.** (**a**) Mid gamma power (maximum ±2.5 Hz mean) correlates with symptom severity within patients both off and on Levodopa. (**b**) Probability of detecting oscillatory peaks across frequency bands using *specparam*. (**c**) Aperiodic broadband power (2–60 Hz) correlates with symptom severity in both conditions. (**d**) Spectra of five representative patients in the Levodopa on condition. Symbols correspond to those in (a) and (c). BR: Bradykinesia-rigidity subscore for each hemisphere.

### Statistical analysis

Throughout the paper, we used an alpha level of 0.05 as a threshold for statistical significance and applied Bonferroni correction where appropriate. In figures and tables, p-values are abbreviated using asterisks (p<0.05: ‘∗’*,* p<0.01*: ‘∗∗’****,*** p<0.001*: ‘∗∗∗’*) if they fall below the Bonferroni-corrected alpha level.

### Dataset comparison

Cohort characteristics (sex, age, disease duration, PD onset age, motor symptoms) were compared across datasets using the non-parametric Kruskal–Wallis test. For variables with significant differences, post-hoc Dunn's test identified the specific dataset pairs and direction of the differences.

### Levodopa modulation of spectra

Standard error for all spectra was calculated through 1000 bootstrap iterations. Differences in spectra between the Levodopa off and on conditions were assessed using the PTE-stats toolbox (github.com/richardkoehler/pte-stats/tree/paper-moritz-gerster), employing non-parametric permutation testing with 1,000,000 permutations at an alpha level of 0.05. Cluster-based corrections were applied to adjust for multiple comparisons when significant p-values were detected, following the approach described by Maris & Oostenveld.^38^ Permutations were conducted by randomly reassigning conditions within patient pairs, with the mean difference used as the test statistic to compare the original and permuted datasets.

### Levodopa modulation of canonical frequency bands

Levodopa off-on effect sizes were calculated using Cohen's *d* based on the mean band power. Cohen's *d* confidence intervals were calculated using 10,000 bootstrap iterations. Using an alpha level of 0.05, we applied the Bonferroni correction to adjust for multiple comparisons of six frequency bands, leading to a confidence level of 1−α/n=1−0.05/6=0.992. Frequency bands were significantly modulated when the 99.2% confidence interval did not cross zero.

### Univariate band correlations across patients

If not indicated otherwise, correlations are computed using Spearman's correlation. Correlation coefficient confidence intervals are calculated non-parametrically using 10,000 bootstrap iterations. Sample size estimations for correlation coefficients were computed based on Cohen (1988).^39^ Correlations with p-values below 0.05 were significant. Multiple comparison corrections were not applied to correct for multiple analysis paths in the multiverse analysis^40^ in Part 1 because the goal of such analysis is to compare statistical results across multiple analysis paths, independent of the number of tested analysis paths. For the same reason, comparisons between spectral frameworks in Part 2 were also not corrected. However, we did apply Bonferroni correction to multiple correlations tested within spectral frameworks to account for multiple frequency bands and multiple Levodopa conditions.

### Frequency correlations across patients

Correlations between motor symptoms and spectral power at individual frequency bins were not Bonferroni-corrected due to the strong dependence of neighbouring frequency bins. Correlations with p-values below 0.05 were considered significant to check their overlap with canonical frequency bands.

### Multivariate band correlations across patients

We analysed the relationship between STN band powers and motor symptom severity by predicting the UPDRS-III scores (y-variable) using linear regression:

$$y_{i}=b_{0}\;+\;b_{1}\;x_{i1}\;+\;...\;+\;b_{p}\;x_{ip}\;+\;\epsilon_{i}$$

where i=1,…,n corresponds to each patient, b are the regression coefficients, p is the number of predictors (band powers), and $\epsilon_{i}$ is the error term. In matrix notation, this simplifies to:

y=Xb+ϵ

The regression coefficients b were calculated by minimising the residuals:

ϵ=y−Xb

To evaluate model fits, we used Pearson's correlation for best comparability with previous studies.

Non-nested linear regression models were compared using J-tests.^41^ Additionally, we calculated the Akaike Information Criterion (AIC) and Bayesian Information Criterion (BIC) using the *statsmodels.api.OLS* function. To account for potential overfitting in models with small sample sizes n, we applied the corrected AIC^42^

$$\text{AI}C_{\text{corrected}}=\text{AIC}\;+\;\frac{2k^{2}\;+\;2k}{n\;-\;k\;-\;1}$$

where k is the number of parameters.

### Within-patient analysis

Within-patient correlations are visualised using repeated measures correlation^43^ and non-parametrically evaluated using ranked repeated measures correlation.^44^ Direct comparison of power between more and less affected hemispheres (Supplementary Fig. S8) was tested using the Wilcoxon signed-rank test. We consider the within-patient analysis in Part 3 of the manuscript as exploratory. Therefore, we did not apply corrections for multiple comparisons when evaluating within-patient correlations across various spectral features.

### Role of funders

The funding sources did not influence the study design, data collection, analysis, or interpretation.

## Results

### Part 1: multicentre reproducibility

In Part 1, we evaluated the reproducibility of Levodopa effects and beta–symptom correlations across multicentre datasets.

#### Dataset comparability

Patient characteristics by dataset (sex, age, disease duration, motor symptoms) are shown in Fig. 2a. Sex, age, disease duration, and bradykinesia-rigidity subscores were comparable. In contrast, off-state UPDRS-III and tremor scores differed (p=0.004 and p=0.0001, respectively). Post-hoc Dunn's test revealed higher UPDRS-III scores for London compared to Berlin and Düsseldorf 1. Tremor was more severe for London compared to Berlin and Düsseldorf 1, and more severe for Düsseldorf 2 compared to Berlin and Düsseldorf 1 (Supplementary Table S3). DBS lead positions were similar along the x-axis but differed in y- and z-axes (Supplementary Fig. S1b).

#### Levodopa modulation of relative spectra

Beta-based aDBS was partly motivated by the observation that Levodopa reduces beta power,^1^^,^^45^ but the consistency of this effect across datasets remains unknown. To assess its consistency, we examined subthalamic power spectra. Fig. 2b shows an exemplary spectrum for relative (i.e., normalised) power in the Levodopa off and on conditions. We studied relative power in our reproducibility analysis to align with previous reports (Supplementary Table S2). We applied Cohen's d to grand mean spectra (Fig. 2c) to quantify Levodopa modulations, defining effect size magnitudes greater than |d|>0.3 as moderate.^46^ We found at least moderate Levodopa-induced enhancements in the theta band in 4/5 datasets, reductions in low beta in 3/5, and reductions in high beta in 2/5 datasets.

While canonical frequency bands facilitate cross-study comparisons, they were primarily defined for cortical EEG and may not optimally capture STN oscillations. To address this, we applied non-parametric cluster-based permutation tests. When pooling all datasets, Levodopa increased power from 3 to 9 Hz (theta) and decreased power from 12 to 29 Hz (beta, Fig. 2c, Table 2). While confirming Levodopa-induced reductions in beta, we also observed strong theta enhancements. Because spectral normalisation enforces interdependencies between bands, the observed theta increase may reflect a mathematical consequence of beta suppression rather than a physiological effect. To disentangle these effects, we analyse absolute power in Part 2.

**Table 2:** Effect of Levodopa on STN power across datasets (Cohen's *d* effect sizes).

Band	Freq. range	Berlin	London	Düsseldorf 1	Düsseldorf 2	Oxford	All datasets
Delta	2–4 Hz	−0.21	0.10	−0.04	**↓ 0.36∗ (2**–**3 Hz)**	**↓** −0.43 **(3**–**4 Hz)**	−0.12
Theta	4–9 Hz	**↑** −**0.54∗ (3**–**8 Hz)**	**↑** −0.52 **(5**–**9 Hz)**	**↑** −**0.38∗ (4**–**7 Hz)**	−0.23 **(6**–**9 Hz)**	**↑ −0.59∗ (6**–**7 Hz)**	**↑ −0.39∗ (3**–**9 Hz)**
Alpha	9–13 Hz	0.12	−0.16	−0.04	−0.11	0.05	0.00
Low Beta	13–20 Hz	**↓ 0.56∗ (11**–**24 Hz)**	**↓ 0.66∗ (13**–**17 Hz)**	0.15	**↓ 0.43∗ (13**–**30 Hz)**	0.15	**↓ 0.41∗ (12**–**29 Hz)**
High Beta	20–30 Hz	0.20	0.14	0.20 **(23**–**35 Hz)**	**↓ 0.31∗ (13**–**30 Hz)**	**↓** 0.39	**0.23∗**
Low Gamma	30–45 Hz	0.01	**↑** −**0.51∗ (34**–**39 Hz, 41**–**43 Hz)**	0.18	0.02	−0.02	−0.01

Corresponding to Fig. 2c. In OFF minus ON calculations, negative values indicate Levodopa-induced power increases (**↑)**, while positive values indicate reductions (**↓)**. Asterisks (∗) denote Bonferroni-corrected significant effect sizes (p<0.008). Arrows (**↑↓)** highlight at least moderate effect sizes |d|>0.3, independent of dataset sample size. Frequency ranges in parentheses indicate clusters of significant modulation.

#### Reproducibility of beta vs. motor symptom correlations

Although Levodopa reduces relative beta power, its pathophysiological relevance depends on its correlation with motor symptoms, which we test next.

To assess the impact of methodological choices, we conducted a multiverse analysis,^40^ which tests a hypothesis across multiple analysis paths. We varied three key factors: 1) Levodopa state (off, on, or off-on difference); 2) sampling strategy (patients or hemispheres); and 3) beta frequency range. To ensure consistency with prior studies, we selected the three most commonly examined beta ranges (Supplementary Fig. S2a): alpha-beta (8–35 Hz), beta (13–30 Hz), and low beta (13–20 Hz). These key factors yielded 18 distinct analyses (3 × 2 × 3).

To assess reproducibility, we examined replicability (consistency across datasets) and robustness (methodological consistency). Here, we present results for the Levodopa off-state and sampling patients (left and right STNs averaged). Results for hemisphere sampling and other Levodopa states are shown in the supplementary material (Supplementary Figs. S2–S4).

Fig. 2d presents Spearman correlations between relative beta power and motor symptoms. Across datasets, the alpha-beta band did not correlate with symptom severity. Significant beta and low beta correlations emerged only in the London dataset, whereas all other datasets failed to reach significance. When pooling all datasets, only low beta power correlated with motor symptoms ($\rho_{L\beta }=0.26$, $p=5e^{-3}$).

A power analysis indicated that at least n=116 patients are needed to replicate this correlation with 80% statistical power. These findings suggest that beta–symptom correlations require large cohorts (n>100), precise frequency definitions (13–20 Hz), and that the relationship is weak (r<0.3),^47^ prompting the question of whether additional spectral features could improve the explained variance.

#### Opposing beta and theta correlations with motor symptoms

To investigate possible spectral correlates of motor symptoms also beyond beta band frequencies, we split patients into ‘severe’ and ‘mild’ groups based on median UPDRS-III symptom scores (Fig. 2e). Patients with severe symptoms showed significantly elevated low beta power (14–17 Hz).

Next, we examined correlations between power and motor symptoms across all frequencies from 1 to 45 Hz using all patients (Fig. 2f). Significant negative correlations (p<0.05) were found in delta (1–2 Hz) and theta (5–8 Hz), while positive correlations appeared in low beta (14–18 Hz) frequencies.

Low beta correlates positively, while lower frequencies correlate negatively with motor symptoms. Because spectral normalisation enforces dependencies between frequency bands, relative power may confound beta–symptom correlations and Levodopa-induced modulations. To address this, we compare spectral frameworks that normalise power, retain absolute values, or separate periodic from aperiodic components next.

### Part 2: spectral framework comparison

Spectral analysis frameworks differ in how neural power is quantified and interpreted. We compared three approaches defined by two distinctions: relative vs. absolute power and total vs. parameterised power. Relative power expresses each band as a percentage of total spectral power (5–95 Hz), while absolute power retains physical units ($\mu V^{2}/Hz$). Total power combines periodic and aperiodic components, whereas parameterisation separates them, distinguishing oscillatory peaks from non-oscillatory broadband activity. We thus evaluated three frameworks: relative total power, absolute total power, and absolute parameterised power. Relative parameterised power was omitted as normalisation eliminates the broadband differences that parameterisation aims to model.

#### Absolute power reveals stronger levodopa modulation of theta than beta

Before examining symptom associations, we assessed Levodopa-induced modulations using absolute power to isolate band-specific changes.

Most previous studies (25 out of 35; Supplementary Table S2) analysed *relative* power, which can complicate interpretation by conflating changes across frequency bands and masking broadband effects. For example, Fig. 3a demonstrates how absolute spectra retain Levodopa-induced broadband power modulations (off: $13.8\;\mu V^{2}$ vs. on: $1.7\;\mu V^{2}$), which are masked in relative power (Fig. 2b, same patient), as normalisation equalises the total to 100%.

Simulations (Fig. 3b) further illustrate these distortions. An increase in absolute theta power reduces *relative* beta power, despite unchanged *absolute* beta (Case 1). Similarly, narrowing the beta peak width lowers *absolute* beta band power but leaves *relative* beta power unaffected and introduces a spurious peak increase (Case 2). These examples illustrate that absolute power more closely reflects genuine band-specific changes.

Analysing absolute spectra (Fig. 3c, Table 3), we observed that Levodopa significantly increased delta and theta power and decreased low beta power. Notably, high beta power remained unaffected. Effect sizes in absolute power were smaller than in relative power, where opposing modulations (theta increase vs. low beta decrease) exaggerated differences (Fig. 3b, Case 1). Crucially, absolute power revealed that Levodopa modulation was stronger in theta than in low beta frequencies (Table 3).

**Table 3:** Effect of Levodopa on STN power across spectral frameworks (Cohen's d effect sizes).

Band	Frequency range	Relative total	Absolute total	Absolute periodic
Delta	2–4 Hz	−0.12	**↑** −**0.17∗**	**↑** −**0.17∗**
Theta	4–9 Hz	**↑** −**0.39∗ (3**–**9 Hz)**	**↑** −**0.26∗ (3**–**9 Hz)**	**↑** −**0.28∗ (2**–**8 Hz)**
Alpha	9–13 Hz	0.00	−0.02	0.08
Low Beta	13–20 Hz	**↓ 0.41∗ (12**–**29 Hz)**	**↓ 0.15∗ (13**–**17 Hz)**	0.18 **(12**–**17 Hz)**
High Beta	20–30 Hz	**↓ 0.23∗**	0.02	0.03
Low Gamma	30–45 Hz	−0.01	−0.03	−0.04 (37–45 Hz)
1/f exponent	2–60 Hz	–	**-**	**↑** −**0.15∗**
Offset	2–60 Hz	–	**-**	**↑** −**0.11∗**
Aperiodic broadband power	2–60 Hz	–	**-**	0.03

In OFF minus ON calculations, significant negative values indicate Levodopa-induced power increases (**↑)**, while positive values indicate reductions (**↓)**. Asterisks (∗) denote Bonferroni-corrected significant effect sizes (p<0.008 for relative and absolute, p<0.006 for periodic). Frequency ranges in parentheses indicate clusters of significant modulation.

#### Total theta power negatively correlates with symptom severity

We next examined absolute power for associations with motor symptom severity. Strongly affected patients exhibited slightly elevated low beta ($d_{L\beta }=+0.23$) and reduced theta power ($d_{\theta }=-0.26$), though effect sizes were not significant (Fig. 3c, right). Correlation analysis (Fig. 3d) showed an overall predominance of negative correlations, suggesting a prokinetic role of broadband power, with significant negative low-frequency (1–11 Hz) and beta-gamma (26–35 Hz) correlations. The strongest correlations were observed for theta ($r_{\theta }=-0.31$, $p=5e^{-4}$), while low gamma showed a negative but non-significant association ($r_{L\gamma }=-0.16$, p=0.076), and low beta was not significantly correlated ($r_{L\beta }=-0.04$, p=0.671). A multiple linear regression model including theta, low beta, and low gamma as predictors yielded a stronger correlation ($r_{\text{Lin}.\;\text{reg}.\;\left(\theta ,L\beta ,L\gamma \right)}=0.42$, $p=1e^{-4}$).

#### Levodopa reduces low beta oscillations and increases theta oscillations

Conventional spectral analysis conflates periodic and aperiodic components. We applied *specparam*^34^ to separate these components (Fig. 3e). Fig. 3f illustrates the advantages of parameterising spectra, where a *larger* offset (case 1) increases absolute and decreases relative band power despite constant periodic band power. A *smaller* 1/f exponent has the same effect (case 2).

Analysing absolute periodic power revealed that Levodopa significantly increased delta and theta oscillations and decreased low beta oscillations. In contrast, high beta oscillations remained unchanged (Fig. 3g). Additionally, the 1/f exponent and offset showed small but significant modulations (Table 3). These findings suggest that Levodopa-induced changes in theta and low beta power reflect true oscillatory modulations while high beta oscillations remain unaffected.

#### Aperiodic offset shows the strongest symptom correlation

To disentangle periodic and aperiodic contributions to symptom severity, we split patients based on median UPDRS-III scores (Fig. 3g, right). Low beta power was higher in severe patients ($d_{L\beta }=0.27$), while theta power was lower ($d_{\theta }=-0.29$), though neither effect reached significance. Correlation analysis (Fig. 3h) confirmed prokinetic theta (4–7 Hz), antikinetic low beta (13–18 Hz), and prokinetic low gamma (29–45 Hz) oscillations. While low beta and low gamma (anti-)correlated with symptoms, the strongest association was observed for the aperiodic offset ($r_{\text{Offset}}=-0.32$, $p=4e^{-4}$), which explains the negative correlation bias in absolute total power (Fig. 3d).

A combined regression model incorporating offset, low beta, and low gamma improved correlation strength ($r_{\text{Lin}.\;\text{reg}.\;\left(\text{Offset},L\beta ,L\gamma \right)}=0.47$, $p=1e^{-4}$). The absolute total theta power used in the regression in Fig. 3d likely reflects both periodic theta oscillations and the aperiodic offset, given their strong correlation (r=0.91, $p=6e^{-48}$) (not shown).

#### Expanding beyond beta improves correlations with symptoms

We built three linear regression models to assess how well relative total, absolute total, and absolute periodic and aperiodic power predict motor symptoms. Because relative power conflates various frequency bands, we included only total relative low beta power as a predictor. To determine whether adding additional frequency bands improves symptom modeling, we incorporated theta, low beta, and low gamma power into the absolute total model (as in Fig. 3d) and the aperiodic offset, periodic low beta, and periodic low gamma power into the absolute periodic model (as in Fig. 3h). To ensure comparability, we used a matched patient set across all models (Fig. 4a), as four patients were excluded from the *specparam* analysis (Materials and methods).

Performance was evaluated using J-tests, alongside corrected Akaike Information Criterion (AIC) and Bayesian Information Criterion (BIC), which assess model fit while penalising complexity. The absolute and periodic models (three predictors each) significantly outperformed the single-predictor relative low beta model ($p_{\text{Abs}>\text{Rel}}=2e^{-4}$, $p_{\text{Per}>\text{Rel}}=2e^{-5}$, Fig. 4b), and the periodic model outperformed the absolute model ($p_{\text{Per}>\text{Abs}}=0.03$4). Furthermore, AIC and BIC values were lowest for the periodic model, followed by the absolute model, and highest for the relative model. Linear mixed-effects models showed that sex, age, disease duration, PD onset age, and the number of days between surgery and recording did not confound the reported associations (Supplementary Material).

Methodologically, these findings indicate that the periodic power framework based on absolute units best models symptom severity. Clinically, the results suggest that motor symptom severity is better explained when low beta power is complemented with low-frequency (aperiodic offset or total theta) and low gamma activity.

### High beta oscillations co-localise with the DBS sweet spot

Beyond symptom correlations, beta power in the STN has been proposed for guiding DBS contact selection.^31^^,^^36^^,^^48^, ^49^, ^50^, ^51^, ^52^ Here, we evaluated which spectral framework best localises the DBS sweet spot using beta power.

We localised relative low and high beta power in the Levodopa off and on conditions (Fig. 5a). To assess spatial alignment, we correlated the localised beta power with the distance to the DBS sweet spot^30^ (blue sphere). Relative low beta power showed no significant correlation, while relative high beta power correlated negatively in the off-condition, indicating closer proximity to the sweet spot. These findings align with Darcy et al.,^31^ who performed the same analysis in an independent cohort.

Repeating the analysis using absolute total and absolute periodic power (Fig. 5b–c) revealed consistent negative correlations across beta bands, Levodopa conditions, and both frameworks.

To compare frameworks, we performed one-tailed non-parametric permutation testing (Fig. 5d). Although absolute and periodic frameworks had comparable correlations, periodic high beta power correlated significantly more strongly with sweet spot distance than relative high beta power (p=0.035). The top right panel of Fig. 5c highlights the close spatial overlap between localised periodic high beta power and the sweet spot, suggesting that beta-based DBS contact selection may be most effective using the periodic power framework.

### Part 3: within-patient correlations

Until now, the present analyses focused on across-patient correlations in the Levodopa off-state. Critically, aDBS biomarkers must track symptoms *within* patients and *across* medication states. To explore this, we treated recordings from individual hemispheres in patients as repeated measures, motivated by the asymmetry of motor symptoms typical in PD. We then inspected whether spectral features on either side correlated with lateral assessments of clinical severity.

Fig. 6a presents conceptual examples of features showing strong across-patient but weak within-patient correlations and vice versa. We included patients with recordings from both STNs and consistent symptom asymmetry across medication conditions to exclude Levodopa-induced side effects (Fig. 6b). We defined symptom asymmetry as a ≥1 point difference between left and right bradykinesia-rigidity subscores. Relative low beta power showed no within-patient correlation (Fig. 6c).

#### Aperiodic broadband power reflects Parkinson's disease severity

Comparing absolute spectra between hemispheres using paired cluster-based permutation testing revealed elevated broadband power in the more affected hemisphere in both Levodopa conditions. In the off-state, the increase spanned significant clusters from 8–13 Hz and 28–60 Hz, extending to 132 Hz (not shown). In the on-state, the shift was significant from 23 to 190 Hz (Fig. 6d, top). Separating periodic and aperiodic components demonstrated that this broadband shift primarily reflected aperiodic power (off: 6–60 Hz; on: 10–60 Hz, Fig. 6f, top), with only minor contributions from periodic oscillations (off: 8–9 Hz and 29–30 Hz; on: 25–33 Hz and 43–45 Hz, Fig. 6e, top). Normalisation removed the broadband shift (Supplementary Fig. S7b top).

Repeated measures correlations showed that absolute total and aperiodic power correlated with the combined bradykinesia-rigidity subscore across broad frequency ranges in both Levodopa conditions. In contrast, periodic power did not (Fig. 6d–f, middle). Among canonical frequency bands, absolute mid gamma power (45–60 Hz) correlated strongest (Fig. 6d, bottom), while no periodic band correlated (Fig. 6e, bottom). Aperiodic parameters showed a significant negative correlation for the 1/f exponent and a positive trend for the offset (Fig. 6f, bottom), aligning with broadband power elevation (Fig. 3f). Summing aperiodic power from 2 to 60 Hz, which we term ‘aperiodic broadband power’, yielded the strongest within-patient correlations (off: $r_{\text{rank}\;\text{rm}}=0.41$, p=4e−4*;* $r_{\text{rank}\;\text{rm}}=0.42$*,* p=0.003; Fig. 6f, bottom).

#### Total mid gamma power: a practical candidate for aDBS

An aDBS biomarker must be symptom-relevant at the individual level and readily extractable in real time. Although aperiodic broadband power strongly correlates with symptoms (Fig. 7c), its extraction requires parameterisation, limiting its immediate clinical use. Instead, total mid gamma power can be rapidly obtained using simple ±2.5 Hz spectral means, as implemented in current aDBS devices (e.g., Medtronic Percept™ PC).

Using a maximum-centered ±2.5 Hz spectral mean, absolute mid gamma power remained significantly correlated with symptoms in both conditions (off: $r_{\text{rank}\;\text{rm}}=0.38$, p=9e−4; on: $r_{\text{rank}\;\text{rm}}=0.38$, p=0.008; Fig. 7a). When tremor scores were added to the bradykinesia-rigidity measure of lateralized motor severity, correlations remained significant. Tremor-only scores showed no significant association, though interpretation is limited by the markedly reduced effective sample size after excluding patients with identical tremor scores on both sides (Supplementary Fig. S10). Many STNs lacked beta peaks even without medication (low beta: ∼50%; high beta: ∼30%; Fig. 7b), limiting their biomarker reliability. Only when considering the full alpha-beta range (8–35 Hz) did at least 88% of STNs have a peak (Supplementary Fig. S7c), as previously shown.^31^^,^^53^^,^^54^

The repeated measures scatter plots (Fig. 7a and c) highlight five representative patients whose spectra in the Levodopa on condition consistently show elevated broadband power in the more affected hemisphere (Fig. 7d). These findings suggest that total mid gamma power captures aperiodic broadband power and has potential as an aDBS biomarker.

## Discussion

We analysed subthalamic nucleus (STN) local field potential (LFP) recordings from patients with Parkinson's disease (PD), emphasising the need for large, heterogeneous cohorts to improve research generalizability. Among three spectral frameworks, parameterised absolute spectra provided the most direct neurophysiological insights and explained motor symptom variance best. Moreover, our findings indicate that aperiodic broadband power may serve as a within-patient biomarker for symptom severity.

In part 1, we performed a reproducibility analysis. Reproducibility challenges in neuroscience often arise from limited statistical power,^55^^,^^56^ methodological variability,^57^^,^^58^ unpublished analysis code,^59^ and cohort homogeneity.^60^ While previous studies linked STN beta power to motor symptom severity, inconsistencies across reports raised the need to study robustness and replicability. Therefore, we conducted a multicentre STN-LFP comparison to evaluate these relationships across five independent datasets.

Previous studies established that Levodopa medication reduces beta power and that beta power scales with motor symptom severity. We successfully replicated these claims in a large dataset. However, significant variability across datasets challenges replicability. Despite typical cohort sizes ranging from 14 to 50 patients, statistical outcomes varied considerably.

We assessed the beta–symptom correlation using three frequency bands, two sampling strategies, and three medication states (off, on, off-on improvement), totalling 18 analyses per dataset. Given the lack of consensus in previous studies on the optimal analysis method (Supplementary Table S2), we applied this multiverse approach^40^ to evaluate robustness (methodological consistency) and replicability (consistency across datasets). With five datasets, this resulted in 90 total tests (five datasets × 18 analyses each). We found only five significant positive correlations, five significant negative correlations, and 80 nonsignificant results (Fig. 2d, Supplementary Figs. S2–S4). Notably, the five positive correlations emerged from four different analysis methods, indicating that cohort differences had a stronger influence on outcomes than methodological differences.

This raises the question of whether cohort variability stems from random sampling error or systematic dataset differences. While datasets are heterogeneous regarding neurosurgery, recording setup, and patient characteristics, none displayed atypical attributes compared to prior studies (Fig. 2a, Supplementary Table S1, Supplementary Fig. S1). More importantly, sex, age, disease duration, PD onset time, and bradykinesia-rigidity symptoms did not differ significantly across datasets (Supplementary Table S3). Differences in total UPDRS-III and tremor scores were observed but can be partly explained by the stun effect, which lowers post-operative UPDRS scores (Supplementary Fig. S5a) without altering STN power–symptom correlations (Supplementary Fig. S5d). While UPDRS assessment involves some subjectivity, it exhibits high inter-rater reliability among trained clinicians.^61^^,^^62^

Some variation, such as DBS lead model and localisation, administered Levodopa dose, and unmeasured factors like race or socioeconomic status, cannot be fully controlled. We are unaware of evidence that these factors systematically influence STN power–symptom correlations, but their impact cannot be entirely excluded. Crucially, follow-up analyses confirmed that sex, age, disease duration, PD onset age, and the number of days between surgery and recording did not confound the reported correlations (Supplementary Material). These findings favour random sampling error over systematic dataset differences as the more likely explanation for divergent results.

To improve generalizability, we pooled all datasets. While some independently published cohorts differ significantly from each other in age or motor symptom distributions, our pooled cohort covers this broader range and shows no significant differences compared to any single independent cohort (Supplementary Fig. S6, Supplementary Table S4), indicating representative patient sampling.

Pooling improved statistical power, revealing the significant relationship between relative beta power and symptom severity that remained obscured in single datasets. Low beta (13–20 Hz) correlated more consistently than wide-band beta (13–30 Hz) and alpha-beta (8–35 Hz) (Fig. 2d, Supplementary Figs. S2–S4), supporting prior findings that clinical beta-based applications should prioritise a narrower frequency range.^5^^,^^33^ These results suggest that inconsistencies in reported beta–symptom correlations primarily result from insufficient sample sizes rather than fundamental limitations of beta power as a biomarker.

We estimate that at least 116 patients are needed to replicate the correlation between relative low beta power and motor symptoms (ρ=0.26) with 80% statistical power. However, a previous large-scale study reported a lower correlation coefficient,^5^ suggesting 178 patients may be required. Given that prior studies had a median sample size of only 13 patients (Supplementary Table S2), these findings underscore the need for larger cohorts or within-patient analyses, such as long-term streaming data, to improve statistical power.^43^^,^^44^^,^^63^

In our review of prior studies (Supplementary Table S2), 22 out of 35 examined relative total beta power, while only six assessed absolute total, three relative periodic, and four absolute periodic power. We compared these spectral analysis frameworks to determine how their methodological differences impact the detection of Levodopa modulation and motor symptom correlations.

Conceptually, our simulations illustrate that relative power is difficult to interpret due to its dependence on other frequency bands. Empirically, Levodopa-induced absolute theta power changes had a 73% larger effect size than absolute low beta power. The opposing modulations of theta and low beta inflated relative power effect sizes, with relative low beta power exhibiting a 170% larger effect size than absolute low beta power.

Our findings on relative power align with previous reports of Levodopa-induced low beta power reductions,^5^^,^^31^^,^^64^, ^65^, ^66^, ^67^, ^68^, ^69^ theta power increases,^64^^,^^66^^,^^70^, ^71^, ^72^, ^73^ and high beta power reductions.^64^ However, absolute and periodic high beta power showed no Levodopa modulation, implying that the observed relative high beta power reduction is a normalisation artefact, distinguishing low and high beta power.^64^

For symptom prediction, absolute power from theta, low beta, and low gamma bands outperformed relative low beta power alone. While incorporating additional bands could improve relative power models, their interpretation remains challenging due to inherent frequency conflation. In contrast, absolute power provides a simpler interpretation by isolating band-specific effects. Our reported prokinetic roles of theta and low gamma have been described in PD before^74^^,^^75^; however, prokinetic subthalamic theta can also become pathological in tremor-dominant patients with PD^76^, ^77^, ^78^, ^79^, ^80^ or dystonia.^81^, ^82^, ^83^ Overall, theta, low beta, and low gamma carry crucial motor-related information, underscoring the importance of considering additional bands beyond beta to understand PD neurophysiology better.

While absolute power improves interpretability over relative power, parameterisation further enhanced symptom modelling, possibly because periodic and aperiodic components reflect distinct neural processes.^34^ The periodic component of low beta correlated with motor symptoms, consistent with studies isolating periodic beta oscillations in LFPs,^84^, ^85^, ^86^ directly measuring neuronal beta bursting,^77^^,^^80^^,^^81^^,^^87^^,^^88^ and observing worsening motor symptoms during beta-frequency DBS.^89^^,^^90^ However, beta oscillations do not correlate with tremor and thus reflect motor symptoms primarily in bradykinetic-rigid patients.^12^^,^^76^^,^^77^^,^^86^^,^^91^, ^92^, ^93^, ^94^

Aperiodic offset negatively correlated with symptoms, consistent with prior work,^9^^,^^85^ but its interpretation remains uncertain due to its persistent correlation with low-frequency power. Disentangling low-frequency oscillations, such as delta and theta, from the aperiodic component is inherently challenging, as these oscillations often lack distinct spectral peaks at a typical 1 Hz resolution—an effect we previously demonstrated through simulations.^95^ As a result, the specparam algorithm may partially attribute low-frequency oscillatory power to the aperiodic component, thereby inflating the estimated offset. Overall, while spectral parameterisation provides valuable insights, determining optimal fitting parameters is challenging, particularly for STN-LFP data.^95^ Thus, parameterised spectra should be interpreted cautiously, while absolute total power–without model fitting–is more robust and compatible with real-time applications.

Despite these challenges, parameterisation significantly improved a previously reported correlation between high beta power and DBS sweet spot distance.^31^ Interestingly, the DBS sweet spot aligned better with high beta than low beta sources, despite its apparently stronger pathological relevance as indicated by Levodopa modulation (Table 3) and symptom correlation (Fig. 3). High beta likely propagates from the motor cortex to the STN via the hyperdirect pathway,^14^ inducing pathological low beta oscillations.^96^ Thus, high beta power may peak where hyperdirect pathway terminals reach the STN, corresponding to the optimal stimulation site.^97^, ^98^, ^99^, ^100^ LFP activity could, therefore, guide DBS contact selection,^101^ particularly using absolute periodic high beta power. Overall, our results suggest that absolute power better reflects neural dynamics than relative power, rendering spectral normalisation unnecessary. Moreover, parameterising absolute power into periodic and aperiodic components further improves symptom modelling performance and electrophysiological interpretability.

In part 3, we focused on STN-LFP biomarkers. Research on PD biomarkers in the Levodopa on-state is scarce. Moreover, a recent study emphasised the importance of distinguishing across-from within-patient correlations,^63^ as the latter control for inter-patient variability–critical in heterogeneous cohorts.^102^ This motivated our search for an STN-LFP marker that tracks symptoms *within* patients *across* medication states.

Conceptually, we demonstrate that an LFP feature can correlate across but not within patients and vice versa (Fig. 6a). Empirically, this distinction is evident in our results. For example, while absolute theta and the aperiodic offset correlated negatively with PD symptoms across patients (off-state, Fig. 3d, h), their correlation coefficients were insignificant or positive within patients (Fig. 6d, f). Similarly, relative and periodic low beta power correlated positively with symptoms across patients but not within patients (Fig. 6c and e). These discrepancies underscore the need to distinguish between across-patient and within-patient associations and reflect the distinct methodological requirements for population-level vs. individual-level biomarker identification.^63^

A prior within-patient study reported increased relative alpha-beta (8–35 Hz) power in the more affected hemisphere for adjacent bipolar channels in the off-state.^53^ We replicated this for adjacent channels (1–2, 2–3, 3–4; Supplementary Fig. S8) but not for distant bipolar channels (1–3, 2–4) used in aDBS sensing.^6^^,^^32^ Furthermore, this effect was absent in the Levodopa on-state (Supplementary Fig. S8), limiting its clinical applicability.

Our finding of absent within-patient beta correlations aligns with long-term streaming studies failing to predict symptoms based on beta power.^8^^,^^103^^,^^104^ However, a recent multicentre clinical trial on beta-based aDBS received regulatory approval,^7^ reaffirming beta's utility as aDBS biomarker. Furthermore, STN beta activity may propagate between hemispheres, diminishing oscillatory asymmetries and concealing within-patient correlations.

In contrast to beta, absolute total broadband power (up to 200 Hz) correlated strongly with lateralized symptoms within patients, independent of medication. Spectral parameterisation revealed that these broadband elevations primarily reflected aperiodic activity, characterised by larger offsets and smaller 1/f exponents. The 1/f exponent has been hypothesised to indicate excitation-inhibition balance,^105^ but aperiodic activity likely reflects multiple distinct physiological processes.^106^ Despite a high correlation between offsets and 1/f exponents,^107^ we observed inverse correlations with symptoms. Combining these parameters as ‘aperiodic broadband power’ provided stronger within-patient symptom correlations than each parameter alone (Fig. 6f).

Why does the more affected hemisphere exhibit elevated aperiodic broadband power, and what could this signify at the neuronal level? In the cortex, neuronal spiking activity is known to increase LFP power across a wide frequency range, spanning 30–100 Hz,^108^, ^109^, ^110^, ^111^, ^112^, ^113^ up to 200 Hz,^114^, ^115^, ^116^, ^117^, ^118^, ^119^, ^120^ or even the entire broadband spectrum^121^^,^^122^—with the notable exception of the 10–20 Hz beta range.^123^ Although subcortical areas are less studied, similar trends appear in the rat hippocampus (100–600 Hz),^124^ rat STN (30–100 Hz),^11^ human amygdala and hippocampus (2–150 Hz),^125^ and human STN in PD (55–95 Hz).^126^ Applied to our findings, broadband power elevations in the more affected hemisphere may reflect increased STN spiking, consistent with evidence from PD animal models^2^^,^^127^, ^128^, ^129^, ^130^, ^131^, ^132^, ^133^ and human intraoperative microelectrode recordings.^80^^,^^87^^,^^134^^,^^135^ Moreover, Levodopa and STN-DBS partially reverse the elevated STN spiking in PD.^11^

While we confirmed that Levodopa increases 1/f exponents,^11^^,^^136^ aperiodic broadband power remained unchanged (Table 3), distinguishing it from beta power and suggesting they reflect independent neural processes. Moreover, while neuronal beta bursting elevates LFP beta power,^137^^,^^138^ spiking outside bursts does not contribute to LFP beta power,^139^ indicating that both processes are differentially reflected in the LFP. Our results suggest that beta power, likely driven by neuronal bursting, and aperiodic broadband power, likely reflecting non-burst spiking, capture distinct pathological mechanisms that differentially affect motor symptoms.

Therefore, aperiodic broadband power shows promise as a potential aDBS biomarker, possibly complementing beta. However, its extraction requires parameterisation, posing challenges for real-time implementation. In contrast, absolute total mid gamma power, which captures aperiodic broadband activity (ρ=0.93, Supplementary Fig. S11), can be readily integrated into existing aDBS systems like the Medtronic Percept™ PC without additional technological advancements.

However, several limitations temper its clinical applicability. First, the correlations identified here might not be strong enough for clinical use, potentially requiring individualised machine learning models and wearable symptom-tracking devices.^140^ Second, our findings stem from single time-point DBS-off resting-state recordings, leaving its dynamics during active DBS or movement unknown. Third, we focused on spectral power below 60 Hz, although high-frequency oscillations (200–400 Hz) may also reflect PD pathology and could offer complementary or superior biomarker properties.^12^^,^^72^^,^^141^, ^142^, ^143^, ^144^

Future research should assess whether absolute mid gamma power and symptoms co-fluctuate over time, especially in naturalistic settings and during DBS. More broadly, as a likely marker of neuronal spiking, aperiodic broadband power may provide valuable insights for future invasive human LFP studies, where direct spiking measurements are often unavailable.

In summary, Part 1 revealed strongly diverging results across five independent datasets processed through a standardised pipeline, highlighting the need for large samples while acknowledging that some residual methodological differences remain. If such differences meaningfully affect results, single-centre studies using fixed protocols could be more prone to bias and less generalizable. Given these considerations, the within-patient correlations examined in Part 3 are particularly informative: they inherently control for variation in patient characteristics, equipment, surgical approaches, and recording protocols. By spanning multiple sites and procedures, the pooled cohort reduces potential biases and improves generalizability. Part 2's systematic comparison of spectral analysis frameworks ensured that the method applied in Part 3 was optimised to reflect the underlying neural dynamics. Together, these elements underscore the potential of multicentre within-patient analyses using spectral parameterisation for identifying robust and replicable individual-level biomarkers.

## Contributors

Conceptualisation, V.N., G.C.; Methodology, M.G., G.W., R.M.K., B.B., W.-J.N., G.C., V.N.; Software, M.G., T.S.B., G.W., R.M.K., N.D.; Formal Analysis, M.G.; Investigation, C.W., R.M.K., T.O.W., L.R., J.Ha., J.L.B., L.K.F., P.K., K.F., G.-H.S., M.S., D.T., K. A., E.P., H.A., L.Z., J.Hi., A.A.K., E.F., A.S., A.O., V.L., H.T., W.-J.N.; Resources, A.V., G.C., V.N.; Data Curation, M.G., T.S.B., C.W., R.M.K., J.V., T.O.W., M.S., D.T., J.Hi., A.A.K., E.F., A.O., V.L., H.T., W.-J.N.; Writing—Original Draft, M.G.; Writing—Review & Editing, M.G., G.W., T.S.B., R.M.K., J.V., T.O.W., J.Ha., L.K.F., J.Hi., A.A.K., E.F., A.S., A.O., V.L., W.-J.N., G.C., V.N.; Visualisation, M.G.; Supervision, G.W., A.V., W.-J.N., G.C., V.N.; Project Administration, M.G., G.C., V.N.; Funding Acquisition, V.N., G.C., A.V. The following authors could directly access and verify the underlying data: Berlin–M.G., T.S.B., R.M.K., J.V., W.-J.N., London–M.G., T.O.W., A.O., V.L., Düsseldorf 1–M.G., M.S., E.F., Düsseldorf 2–M.G., D.T., J.Hi., Oxford–M.G., C.W., H.T. All authors read and approved the final version of the manuscript.

## Data sharing statement

The Oxford dataset is openly available at https://data.mrc.ox.ac.uk/stn-lfp-on-off-and-dbs, and the Düsseldorf 2 dataset is openly available at https://openneuro.org/datasets/ds004907/versions/1.3.0 and reported in *scientific data.*^25^ For requests regarding the Berlin dataset, please contact Wolf-Julian Neumann (julian.neumann@charite.de) or the Open Data officer (opendata-neuromodulation@charite.de). For London, please contact Vladimir Litvak (v.litvak@ucl.ac.uk), and Esther Florin (esther.florin@med.uni-duesseldorf.de) for Düsseldorf 1.

All analysis code is made publicly available at github.com/moritz-gerster/STN_broadband_power. T.S.B. reviewed the analysis code for correctness.

## Declaration of interests

K.A. received educational grants from Medtronic and Abbott. W.-J.N. received honoraria for consulting from InBrain—Neuroelectronics that is a neurotechnology company and honoraria for talks from Medtronic that is a manufacturer of deep brain stimulation devices unrelated to this manuscript. L.K.F. received honoraria for talks from Medtronic. A.A.K. has served on advisory boards of Medtronic and has received honoraria and travel support from Medtronic, Boston Scientific, and Bial. A.A.K. reports a relationship with Medtronic that includes consulting or advisory, speaking and lecture fees, and travel reimbursement. A.A.K. reports a relationship with Boston Scientific Corporation that includes speaking and lecture fees, and travel reimbursement. P.K. has served on advisory boards for Medtronic, AbbVie and Gerresheimer and received lecture fees from Stadapharm and AbbVie. E.P. received a research grant from Saluda; royalties from Elsevier and Oxford University Press; honoraria for lectures from Boston Scientific, Nevro, and Medtronic; and travel support from Boston Scientific and Medtronic. L.Z. received consulting fees, honoraria for lectures, travel support, and advisory board honoraria from Medtronic and Boston Scientific. A.S. received consulting fees from Metronic, Abbott, and Boston Scientific; and honoraria from BSH Medical Communications, Zambon, and Bial. G.W. received funding from the Deutsche Forschungsgemeinschaft (Project ID 511192033) and is employed part-time at Charité and in a private neurological practice. V.L. received support from Medtronic for attending meetings, and travel (UK aDBS workshop), and a loan of a DBS stimulator for bench testing. J.L.B. received honoraria from Medtronic for lectures and educational activities and served on a Medtronic advisory board. The remaining authors declare no competing interests.

## Appendix A Supplementary data

Supplementary Clean
