# Supplementary material for: Beyond beta rhythms: subthalamic aperiodic broadband power scales with Parkinson's disease severity–a cross-sectional multicentre study
Source: eBioMedicine. 2025 Oct 29;122:105988. doi: 10.1016/j.ebiom.2025.105988 (PMC12790593; doi:10.1016/j.ebiom.2025.105988)
Supplement: Supplementary Clean [file mmc1.pdf]

Supplementary Material

Supplementary Methods 2
Dataset characteristics 2
Software 3
Aperiodic broadband power 3
Linear mixed-effects modelling 3
Supplementary Results 4
Part 1: Multicenter reproducibility 4
Representative studies on the beta-symptom correlation 4
Reproducibility of beta versus motor symptom correlations 6
Stun effect: Pre- versus post-operative UPDRS assessment 9
Dataset comparability 11
Pooled multicenter cohort comparison with previous studies 11
Confounding effects of demographic variables on correlations 12
Part 2: Spectral framework comparison 15
Theta, low beta, and low gamma oscillations explain unique variance of motor symptom severity 15
Part 3: Within-patient correlations 16
Spatial specificity of spectral biomarkers 17
Influence of tremor on within-patient correlations 18
Relationship between mid gamma power and aperiodic parameters 19
Age-related effects on subthalamic spectral features 20
References 22

Supplementary Methods

Dataset characteristics

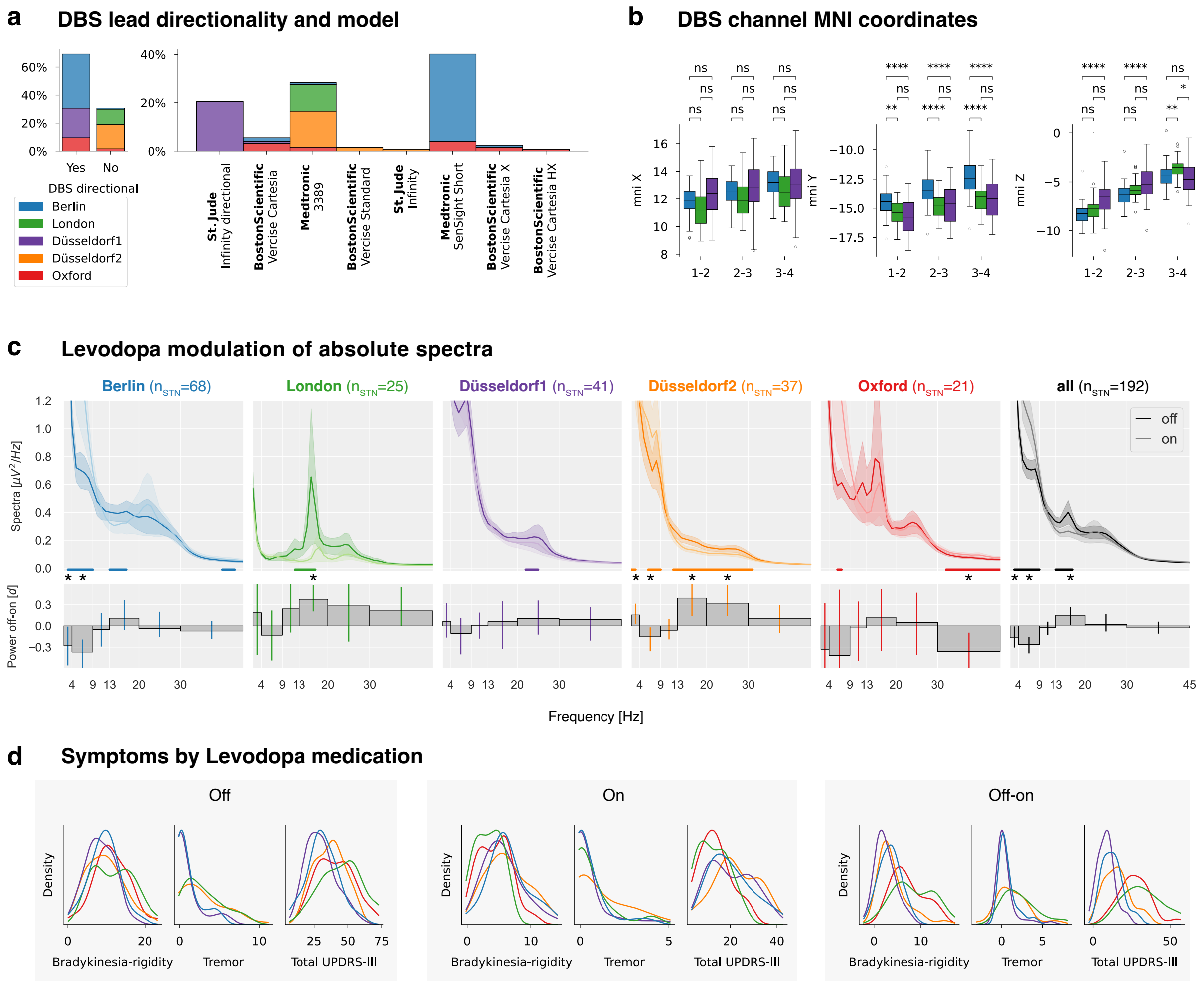

Supplementary Fig. S1 | Dataset characteristics. a, DBS lead models by dataset. **b**, DBS channel localisations for the three datasets with available MNI coordinates. P-values are Bonferroni-corrected for each coordinate with a threshold at $p = 0 \cdot 006$. **c**, Same as Fig. 2c for absolute instead of relative power. **d**, PD symptom characteristics by medication status for all datasets.

Supplementary Table S1 | Dataset surgery and recording details

Dataset	Number of patients (female)	Number of micro recordings during surgery	Sample rate/ highpass/ lowpass	Amplifier	Patients with directional DBS leads	Recording reference	Individual On-state Levodopa dose	Off- and on-state recording on the same day	DBS center	Publications
**Berlin**	50 (12)	2	4096 Hz / - / 1600 Hz	TMSi Saga	98%	Lowermost DBS contact (left or right)	Usual dose	No (off: day 4·3 ± 1·3, on: 3·8 ± 1·5)	Department of Neurosurgery at the Charité – Universitätsmedizin Berlin	1–3
**London**	14 (3)	0	2400 Hz / 1 Hz / 600 Hz	CTF MEG System	0%	Right mastoid	Usual dose	No (day 2 or 3 counterbalanced across patients)	National Hospital of Neurology and Neurosurgery and the University College London Institute of Neurology	4
**Düsseldorf1**	27 (7)	3·4 ± 1·2	2400 Hz / - / 800 Hz	ElektaNeuromag	100%	mastoid	1·5 times the Usual dose	yes	Department of Functional Neurosurgery and Stereotaxy in Düsseldorf	5–10
**Düsseldorf2**	22 (6)	Up to 5	2000 Hz / 0.1 Hz / 660 Hz	ElektaNeuromag	0%	mastoid	1·5 times the usual dose	yes	Department of Functional Neurosurgery and Stereotaxy in Düsseldorf	10–12
**Oxford**	17 (6)	0	2048 Hz (TMSi Porti) or 4096 Hz (TMSi Saga) / - / 1600 Hz	TMSi Porti/Saga	71%	bipolar or common average	Usual dose	yes	St. George's University Hospital NHS Foundation Trust, London (13 patients); King's College Hospital NHS Foundation Trust, London (4 patients)	13–15

Software

Offline processing was performed with custom Python scripts using NumPy,[16] SciPy,[17] MNE-Python,[18] MNE-BIDS,[19] Pandas,[20,21] specparam,[22] seaborn,[23] Pingouin,[24] statsmodels,[25] and PTE-Stats (github.com/richardkoehler/pte-stats/tree/paper-moritz-gerster), as well as custom MATLAB scripts using SPM12,[26] and Lead-DBS.[27]

Aperiodic broadband power

The following code illustrates the extraction of aperiodic broadband power using *specparam*[22] in the Python programming language for 2–60 Hz:

```
from specparam import SpectralModel

# Fit the spectral model and extract the aperiodic power
fm = SpectralModel()
fm.fit(frequencies, power_spectrum, [2, 60])
aperiodic_broadband_power = fm._ap_fit.sum()
```

Linear mixed-effects modelling

We tested whether demographic variables confounded the relationships between STN spectral features and motor symptoms using linear mixed-effects models with STN band power and total UPDRS-III scores as variables of interest. Fixed effects included band power and demographic covariates (age, sex, disease duration, PD onset age, days between surgery and recording). Dataset identity was modelled as a random intercept to account for site-level differences. Models were estimated by restricted maximum likelihood (REML) using the *statsmodels.formula.api.mixedlm* function in Python.

Supplementary Results

Part 1: Multicenter reproducibility

Representative studies on the beta-symptom correlation

To provide context for our multicenter reproducibility analysis, we reviewed representative studies correlating macro STN-LFP resting-state DBS-off beta power with UPDRS-III (sub-)scores. We included correlations from the baseline off-medication condition or off-on modulations of beta power and UPDRS-III improvements (Table S1). We found no studies specifically reporting the baseline on-medication condition. Studies calculating correlations using combined data from off and on conditions were excluded. While not exhaustive, the selected studies illustrate the variability in sample sizes (7 to 103 patients, median 13), methodologies (independent variable: absolute total, relative total, absolute periodic, or relative periodic beta power; dependent variable: lateralised UPDRS-III subscore or total UPDRS-III score), and results (17 significant vs. 22 insignificant correlations). The applied frequency borders for the "beta" band are visualised in Supplementary Fig. S2a. We extracted the three most common frequency borders and referred to them as alpha-beta (8–35 Hz), beta (13–30 Hz), and low beta (13–20 Hz) bands. Studies were listed multiple times if they performed multiple analyses (such as multiple Levodopa medication conditions) to compare them with our multiverse analysis.

Supplementary Table S2 | Representative selection of studies analysing correlations between beta power and motor symptoms using Levodopa off-state macro STN-LFP recordings.

Study	Citation	# patients	# STNs	Correlation	p-value	Band [Hz]	LDOPA	UPDRS items	Aperiodic removed	Relative power [Normalisation range]	post-OP days	Notes
**1**	**Kühn 2006[28]**	**9**	**17**	**0·84**	**< 0·001**	**8-35**	**(Off-On)/Off**	**Contra BR**	**No**	**n/a**	**5·7 ± 0·3**	**STNs without off beta peaks excluded**
**2**	**Ray 2008[29]**	**7**	**11**	**0·7**	**< 0·05**	**8-35**	**(Off-On)/Off**	**Contra BR**	**No**	**n/a**	**n/a**	**STNs without off beta peaks excluded**
3	Ray 2008[29]	7	11	-0·35	0·15	8-35	Off	Contra BR	No	n/a	n/a	STNs without off beta peaks excluded
**4**	**Kühn 2009[30]**	**30**	**51**	**0·62**	**< 0·001**	**8-35**	**(Off-On)/Off**	**Contra BRT**	**No**	**yes (5-45 Hz)**	**3 to 6**	
5	Chen 2010[31]	12	23	0·49	n.s.	13-35	Off	Contra BRT	No	no	5	
6	Chen 2010[31]	12	23	0·51	n.s.	13-35	Off	Contra BRT	No	yes (n/a)	5	
**7**	**López-Azcárate 2010[32]**	**13**	**26**	**0·43**	**0·029**	**12-20**	**Off**	**Contra BR**	**Yes**	**no**	**4 to 5**	**periodic power: ratio peak power/adjacent spectrum**
**8**	**Özkurt 2011[33]**	**9**	**18**	**0·33**	**0·028**	**8-35**	**Off**	**Contra BR**	**No**	**no**	**1**	
9	Hohlefeld 2012[34]	9	18	0·08	0·71	13-35	Off-On	UPDRS-III	No	yes (8-35 Hz)	2 to 6	
10	Little 2012[35]	10	17	LME	n.s.	12-33	(Off-On)/Off	Contra BRT	No	n/a	ca. 5·7	9 of 10 patients from Kühn 2006
**11**	**Trager 2016[36]**	**9**	**n/a**	**LME**	**0·036**	**13-30**	**Off**	**Contra BRT**	**No**	**yes (n/a)**	**0, 6, and 12 months**	**9 TD patients at 0 and 6 months, 4 TD patients at 12-month follow-up, beta and UPDRS negatively correlated for TD patients**
**12**	**Trager 2016[36]**	**8**	**n/a**	**LME**	**0·036**	**13-30**	**Off**	**Contra BRT**	**No**	**yes (n/a)**	**0, 6, and 12 months**	**8 BR patients at 0 and 6 months, 6 BR patients at 12-month follow-up, beta and UPDRS positively correlated for BR patients**
**13**	**Neumann 2016[37]**	**63**	**n/a**	**0·44**	**<0·0001**	**8-35**	**Off**	**UPDRS-III**	**No**	**yes (5-45 and 55-95 Hz)**	**n/a**	**strongest correlation for 10-14 Hz**
**14**	**West 2016[38]**	**12**	**22**	**0·56**	**0·007**	**13-20**	**Off-On**	**Contra BR**	**No**	**yes (4-48 Hz)**	**ca. 2 to 3**	
15	West 2016[38]	12	22	0·37	0·08	21-30	Off	Contra BR	No	yes (4-48 Hz)	ca. 2 to 3	
**16**	**West 2016[38]**	**12**	**22**	**0·66**	**0·001**	**13-20**	**Off**	**Contra BR**	**No**	**yes (4-48 Hz)**	**ca. 2 to 3**	
**17**	**Beudel 2017[39]**	**39**	**76**	**0·4**	**<0·0005**	**13-30**	**Off**	**Contra BR**	**No**	**yes (5-45 and 55-95 Hz)**	**n/a**	**# STNs estimated from plot and p-value**
18	Beudel 2017[39]	39	76	0·23	0·14	10-14	Off	UPDRS-III	No	yes (5-45 and 55-95 Hz)	n/a	# STNs estimated from plot and p-value
19	Beudel 2017[39]	39	76	0·28	0·07	8-35	Off	UPDRS-III	No	yes (5-45 and 55-95 Hz)	n/a	# STNs estimated from plot and p-value
20	Neumann 2017[40]	12	24	n/a	n.s.	13-35	Off-On	Contra BRT	No	yes (5-45 and 55-95 Hz)	3 and 8 months	Clin Neurophysiol. 2017
21	Neumann 2017[41]	11	n/a	-0·21	0·37	8-35	Off	UPDRS-III	No	yes (5-45 and 55-95 Hz)	n/a	Mov Disord. 2017, only TD patients from Neumann 2016 included
**22**	**Neumann 2017[41]**	**11**	**n/a**	**0·93**	**0·0001**	**8-35**	**Off**	**UPDRS-III**	**No**	**yes (5-45 and 55-95 Hz)**	**n/a**	**Mov Disord. 2017, only BR patients from Neumann 2016 included**
23	van Wijk 2017[42]	14	22	0·27	0·222	13-30	Off	Contra BR	No	no	intraoperative	
24	Martin 2018[43]	13	26	ca. 0·4	n/a	13-30	Off	Contra BR	No	no	1 to 2	correlation estimated from Fig. 2b
**25**	**Martin 2018[43]**	**13**	**26**	**0·68**	**0·0004**	**13-30**	**Off**	**Contra BR**	**Yes**	**no**	**1 to 2**	
26	Ozturk 2020[44]	9	9	0·4	0·06	13-22	Off-On	Contra BR	No	yes (120-160 Hz off)	n/a	
27	Eisinger 2020[45]	15	19	0·05	0·85	12-30	Off	Contra B	No	no	n/a	
28	Eisinger 2020[45]	15	19	0·33	0·3	12-30	Off	Contra R	No	no	n/a	
**29**	**Wiest 2020[15]**	**14**	**11**	**0·67**	**0·024**	**13-35**	**Off**	**Contra BRT**	**No**	**yes (5-45 Hz)**	**2 to 5**	
**30**	**Averna 2022[46]**	**7**	**12**	**0·65**	**0·022**	**12-20**	**Off-On**	**UPDRS-III**	**No**	**yes (600-1000 Hz)**	**3**	**Correlation from contact 0-3**
**31**	**Lofredi 2023[47]**	**103**	**n/a**	**0·361**	**0·001**	**13-20**	**Off-On**	**UPDRS-III**	**No**	**yes (5-98 Hz)**	**1 to 7**	**3 patients without UPDRS**
**32**	**Lofredi 2023[47]**	**103**	**n/a**	**0·21**	**0·03**	**13-20**	**Off**	**UPDRS-III**	**No**	**yes (5-98 Hz)**	**1 to 7**	**3 patients without UPDRS scores**
33	Pardo-Valencia 2023[48]	13	21	0·05	n/a	13-35	Off-On	UPDRS-III	Yes	yes (460-490 Hz)	3 to 5	
34	Pardo-Valencia 2023[48]	13	21	0·1	n/a	13-35	Off-On	UPDRS-III	No	yes (460-490 Hz)	3 to 5	
35	Pardo-Valencia 2023[48]	21	33	0·06	n/a	13-35	Off	UPDRS-III	Yes	yes (460-490 Hz)	3 to 5	one-tailed Bayes factor 0·296 -> moderate evidence for absence of correlation
36	Pardo-Valencia 2023[48]	21	33	0·17	n/a	13-35	Off	UPDRS-III	No	yes (460-490 Hz)	3 to 5	one-tailed Bayes factor 0·550 -> anecdotal evidence for absence of correlation
37	Wiest 2023[13]	14	28	-0·04	0·85	13-35	Off-On	Contra BR	Yes	yes (1-90 Hz)	3 to 6	
38	Wilkins 2023[49]	21	42	LME	1	13-30	Off	Contra R	Yes	no	3 to 70 months	
39	Wilkins 2023[49]	21	42	LME	1	13-30	Off	Contra B	Yes	no	3 to 70 months	
	**This study**	**99**	**178**	**0·25**	**0·01**	**13-20**	**Off-On**	**UPDRS-III**	**No**	**no**	**2·7 ± 1·8**	
	**This study**	**94**	**164**	**0·31**	**0·004**	**13-20**	**Off-On**	**UPDRS-III**	**Yes**	**no**	**2·7 ± 1·8**	
	**This study**	**99**	**178**	**0·31**	**0·002**	**13-20**	**Off-On**	**UPDRS-III**	**No**	**yes (5-95 Hz)**	**2·7 ± 1·8**	
	This study	119	216	-0·04	0·69	13-20	Off	UPDRS-III	No	no	2·7 ± 1·8	
	**This study**	**119**	**216**	**0·28**	**0·003**	**13-20**	**Off**	**UPDRS-III**	**No**	**yes (5-95 Hz)**	**2·7 ± 1·8**	
	**This study**	**119**	**216**	**0·24**	**0·01**	**13-20**	**Off**	**UPDRS-III**	**Yes**	**no**	**2·7 ± 1·8**	
**Median**		**13**	**22**									

Significant positive correlations ($p < 0 \cdot 05$) are bold. B: bradykinesia, R: rigidity, Contra BR(T): contralateral bradykinesia-rigidity(-tremor) UPDRS-III subscore, LME: linear mixed effect model.

Reproducibility of beta versus motor symptom correlations

In Supplementary Fig. S2d-f, we averaged band powers of the left and right STNs. To account for the variability in analysis strategies used in previous studies, we performed the same correlations by sampling each STN (instead of each patient) and the corresponding contralateral UPDRS-III subscores for bradykinesia and rigidity (UPDRS-III items 22 to 26, Supplementary Fig. S2c, h-k). On the single dataset level, we observed two significant negative correlations for the Oxford dataset (alpha-beta and beta) and a positive correlation for Düsseldorf2 and the low beta band. The pooled correlation coefficients were insignificant for the alpha-beta and beta bands ($\rho_{\alpha\beta} = 0 \cdot 03, p = 0 \cdot 61$ and $\rho_{\beta} = 0 \cdot 09, p = 0 \cdot 19$) and significant for the low beta band ($\rho_{L\beta} = 0 \cdot 17, p = 0 \cdot 01$). Note, however, that the estimated sample size to achieve proper replicability is $n_{\mathrm{STN}} = 261$ STNs. For this investigation, only $n_{\mathrm{STN}} = 216$ STNs were available.

We considered whether variability in beta peak frequencies across patients and STNs might affect correlations. Specifically, we investigated whether individual peak power, rather than power at fixed frequencies, might carry more meaningful pathological information. We, therefore, correlated the motor symptoms with the extracted maximum band powers. However, the results shown in Supplementary Fig. S2f were similar to those in panel b - with a negative correlation for the delta and theta bands ($p < 0 \cdot 05$) and a positive correlation for the low beta band ($p < 0 \cdot 01$). A scatter plot of the pooled low beta correlation from Fig. 2d is provided in Supplementary Fig. S2g. Note that the mean low beta power correlation ($\rho_{\mathrm{mean}(L\beta)} = 0 \cdot 26, p = 5e^{-3}$) is similar to the maximum low beta power correlation ($\rho_{\max\,(L\beta)} = 0 \cdot 26, p = 0 \cdot 01$). The panels d-g in Supplementary Fig. S2 are also shown for the lateralised analysis in panels h-k. Instead of correlating baseline beta power with baseline UPDRS-III score in the Levodopa off condition, we also correlated the Levodopa modulation of the power in the beta band with the improvement of concomitant motor symptoms in Supplementary Fig. S3. The results for the baseline Levodopa on condition are shown in Supplementary Fig. S4.

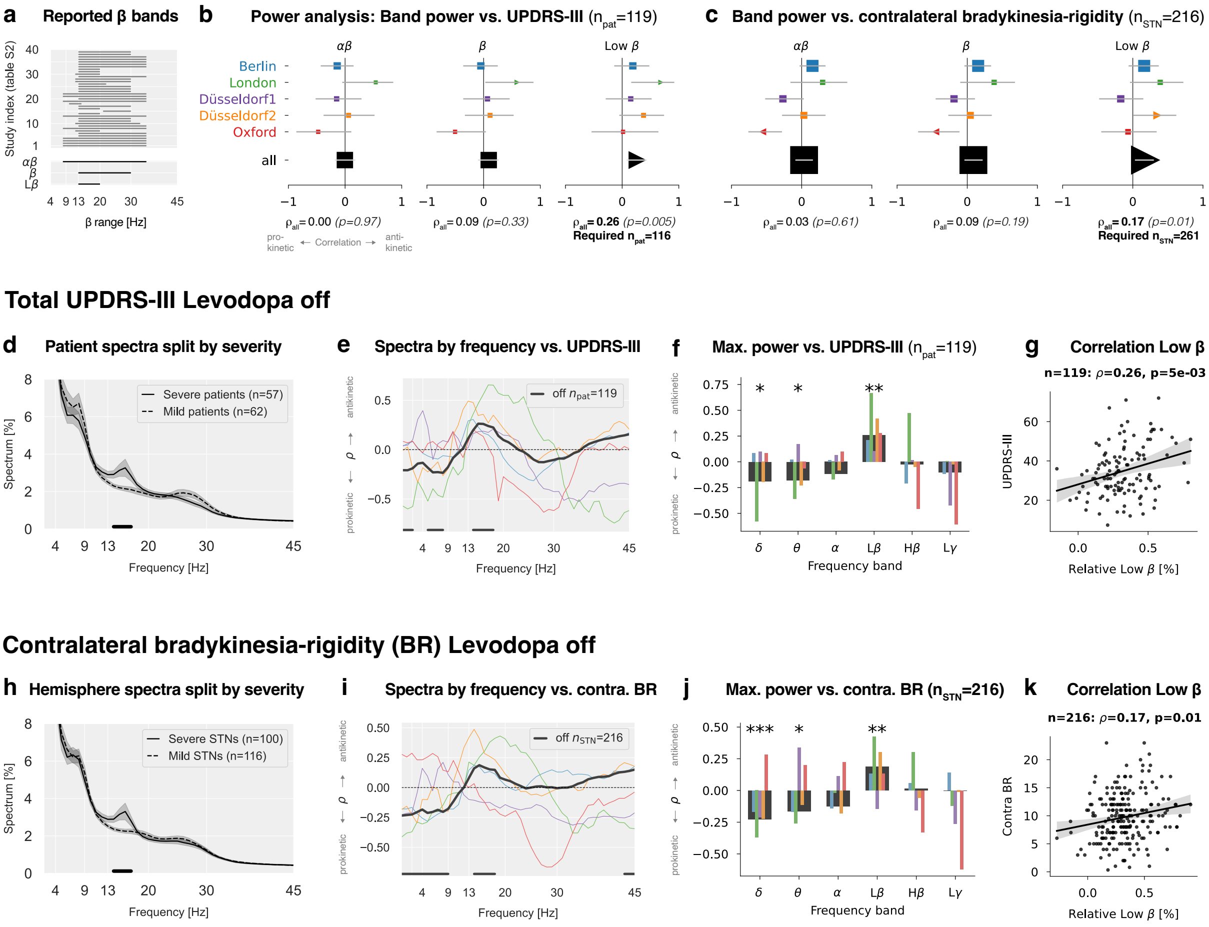

Supplementary Fig. S2 | Association between relative spectral power and motor symptom severity for single and pooled datasets. a, Top: Beta band definitions used in previous studies (Table S2) correlating beta power with PD severity. Bottom: Frequency borders for the alpha-beta (8–35 Hz), beta (13–30 Hz), and low beta (13–20 Hz) bands used in this study. **b**, Correlations between average band power (left and right STNs averaged) and total UPDRS-III scores. X-axis: Spearman's correlation coefficients, y-axis: datasets, horizontal lines: 95th percentile confidence intervals, symbol sizes represent the dataset sample sizes. Markers for non-significant correlations are displayed as squares and significant correlations as triangles. The pooled correlation coefficients and p-values are shown at the bottom. "Required n_{pat}" indicates the sample size estimations to achieve a statistical power of 80% for the observed correlation coefficients. **c**, Same as **b,** treating each STN (left or right) as a single sample and correlating it with the contralateral bradykinesia-rigidity UPDRS-III subscore. **d**, Patient spectra split by their median UPDRS-III score. The horizontal line shows a 14–17 Hz cluster of significant difference. **e**, Correlation between power and UPDRS-III for each frequency bin of the power spectrum. Horizontal lines indicate frequencies with p-values < 0·05 for the pooled data. **f**, Correlations for maximum band power instead of mean band power. **g**, The scatter plot of the strongest pooled correlation from **b**. **h-k**, Same as **d-g;** however, without averaging left and right STNs, the contralateral bradykinesia-rigidity subscore instead of the total UPDRS-III score is used as a target.

Levodopa improvement off-on

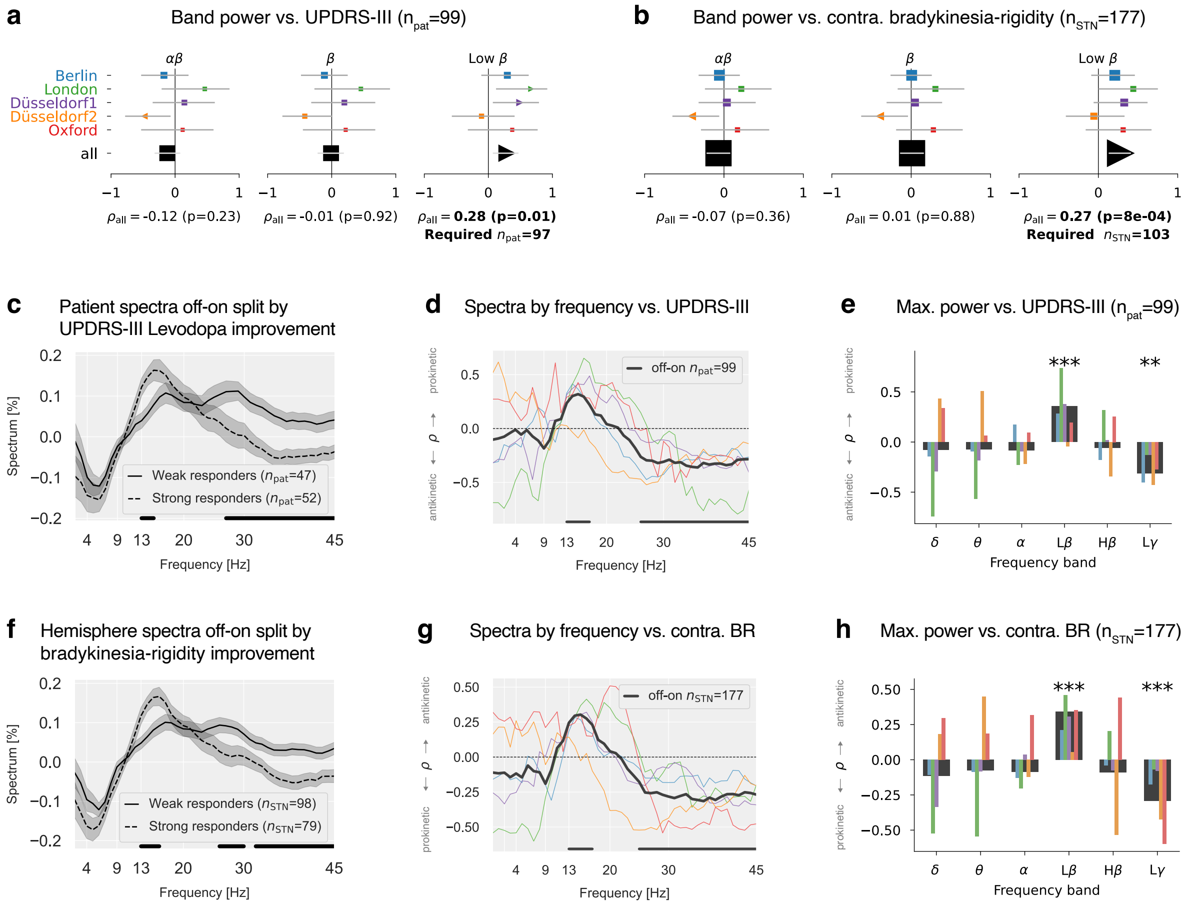

Supplementary Fig. S3 | Same as **Fig.** S2 for the Levodopa off-on improvement instead of the Levodopa off condition. The band power corresponds to the Levodopa-induced change of the power, and the UPDRS-III (sub-)scores correspond to the Levodopa-induced symptom improvement.

Levodopa on

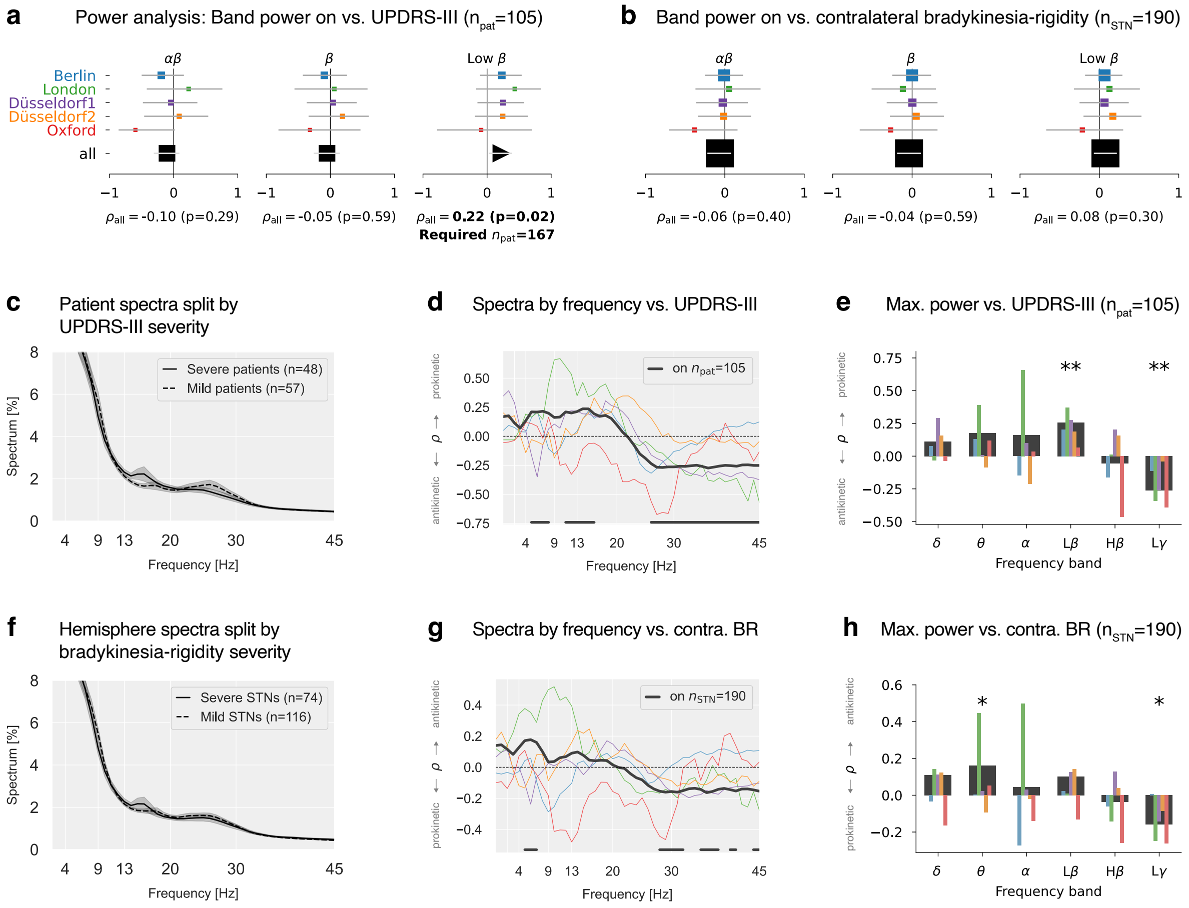

Supplementary Fig. S4 | Same as **Fig. S3** for the Levodopa on condition.

Stun effect: Pre- versus post-operative UPDRS assessment

UPDRS-III assessment was performed only pre-surgically for Oxford and London and only post-surgically for Düsseldorf2, while for Berlin and Düsseldorf1, both assessment time points were available (Fig. 2a). We compared pre- and post-surgical UPDRS-III scores for Berlin and Düsseldorf1 and observed significantly lower scores for the off- and off-on condition (Supplementary Fig. S5a), possibly indicating a stun effect.[50] Moreover, the difference between the pre- and post-UPDRS-III scores (post-pre) correlated negatively with the number of recovery days after surgery for the off-and-on condition, indicating that the stun effect was more pronounced for fewer days between surgery and measurement (Supplementary Fig. S5b).

We further investigated the impact of the stun effect on the LFP power using the Berlin, Düsseldorf1, and Oxford datasets. London and Düsseldorf2 were excluded because, in those datasets, all recordings were performed on a fixed number of days between surgery and recording. Correlating the number of days between surgery and recording with LFP power revealed that low-frequency power (delta, theta, alpha) decreased with more days since surgery. In contrast, low beta, high beta, and low gamma power increased (Supplementary Fig. S5c). This indicates that beta power is suppressed after the surgery and rebounds afterwards. Despite these important factors impacting both the STN spectrum and the UPDRS-III assessment, the correlations for the pre-and post-operative UPDRS-III scores

were similar ($\rho_{L\beta\ \mathrm{off\ pre}} = 0 \cdot 24$, $\rho_{L\beta\ \mathrm{off\ post}} = 0 \cdot 21$, $\rho_{L\beta\ \mathrm{off-on\ pre}} = 0 \cdot 46$, $\rho_{L\beta\ \mathrm{off\ post}} = 0 \cdot 42$, Supplementary Fig. S5d), in line with Pardo-Valencia.[48] These findings suggest that the most realistic UPDRS-III symptom assessment (unaffected by the stun effect) is achieved pre-operatively or many days after the surgery. However, the stun effect appears to reduce beta power and UPDRS-III scores similarly, therefore having no strong impact on the reported correlations. Finally, no surgical microelectrode recordings were performed for the London and Oxford datasets (Table 3), possibly reducing the strength of the stun effect.[51]

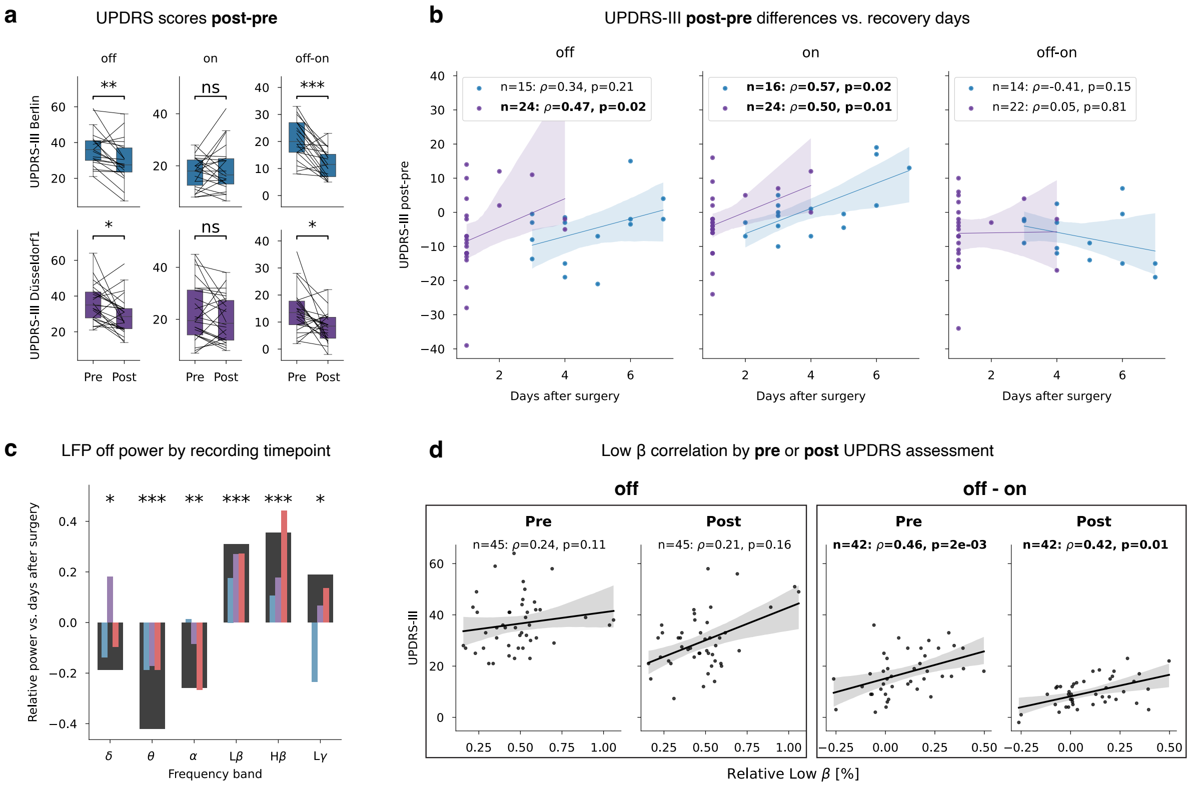

Supplementary Fig. S5 | Stun effect. a, Comparison of pre-and post-operative UPDRS-III scores. Pre- and post-operative scores were available for patients from Berlin and Düsseldorf1 (c.f. Fig. 2a). **b**, The difference in UPDRS-III scores (post - pre) decreases with increasing recovery days after the surgery. Same colours as in **a**. **c**, Delta, theta, and alpha decrease during recovery. Low beta, high beta, and low gamma power increase during recovery. Same colours as in **a**. Red: Oxford. Black: All. We excluded London and Düsseldorf2 from this analysis because all patients' recovery days were equal, prohibiting a correlation analysis (c.f. Fig. 2a). **d**, Comparison of correlating beta power with the pre- vs. post-operative UPDRS-III scores for Berlin and Düsseldorf1 pooled.

Dataset comparability

Supplementary Table S3 | Kruskal-Wallis multicenter cohort comparison.

Cohort characteristic	Kruskal statistic	Kruskal p-value	Post-hoc differences
**Sex**	0·731	0·947	
**Age**	8·914	0·063	
**Disease duration**	10·738	0·030	
**PD onset age**	11·551	0·021	
**UPDRS-III (off)**	**15·561**	**0·004**	London (pre) > Berlin (post) (p=0·025)<br>London (pre) > Düsseldorf1 (post) (p=0·015)
**Bradykinesia-rigidity (off)**	8·138	0·087	
**Tremor (off)**	**20·611**	**0·0001**	London (pre) > Berlin (post) (p=0·010)<br>London (pre) > Düsseldorf1 (post) (p=0·002)<br>Düsseldorf2 (post) > Berlin (post) (p=0·038)<br>Düsseldorf2 (post) > Düsseldorf1 (post) (p=0·006)<br><br>Missing data: Oxford

Multicenter cohort comparison including Berlin, London, Düsseldorf1, Düsseldorf2, and Oxford. Bonferroni-corrected ($n = 7$) p-value threshold for Kruskal-Wallis test: $p = 0 \cdot 007$. Dunn's post hoc p-values are Bonferroni-adjusted. ">" denotes directionality of significant post-hoc differences. Bradykinesia-rigidity and tremor subscores were averaged for left and right sides. "pre"/"post" indicates whether UPDRS was assessed before or after DBS surgery; when both were available, post-operative scores were used. Note that post-operative UPDRS scores are typically lower due to the stun effect.[50]

Pooled multicenter cohort comparison with previous studies

We compared our pooled multicenter cohort with other published cohorts to assess whether pooling heterogeneous datasets produced a representative patient sample.

Five studies with the largest sample sizes in Supplementary Table S2 were selected, excluding Neumann 2016 (due to cohort overlap with Lofredi 2023), Wiest 2020, and West 2016 (overlap with this study's Oxford and London cohorts), and Beudel 2017 and van Wijk 2017 (no published cohort characteristics). The final selection comprised Lofredi 2023 ($n =$ 106, Charité, Berlin, Germany), Wilkins 2023 ($n = 21$, Stanford University Hospital, USA), Pardo-Valencia 2024 ($n = 21$, Hospital Clínico San Carlos, Madrid, Spain), Eisinger 2020 ($n = 15$, University of Florida Norman Fixel Institute for Neurological Diseases, Gainesville, USA), and Martin 2018 ($n = 13$, Lausanne University Hospital, Switzerland). The DBS centre in Lofredi 2023 corresponds to the Berlin centre in our study; however, the patient samples do not overlap.

From each study, we extracted sex, age, disease duration, PD onset age, and UPDRS-III off-state scores. When both pre- and post-operative scores were available, post-operative scores were selected. Eisinger 2020 lacked disease duration and PD onset age; Martin 2018 lacked

total UPDRS-III scores. In all studies, bradykinesia-rigidity and tremor subscores were either unavailable or defined inconsistently and therefore not compared.

No significant differences were found between this study's pooled cohort and any other study cohort (Supplementary Fig. S6, Supplementary Table S4).

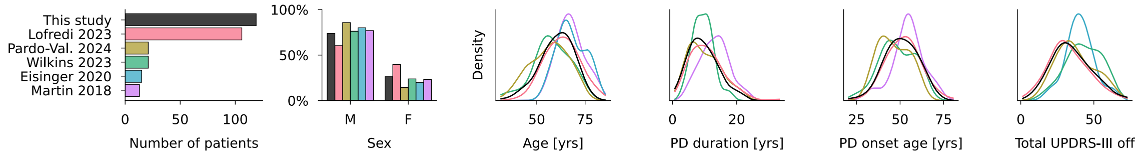

Supplementary Fig. S6 | Multi-study cohort comparison. Number of patients per study; sex distribution; kernel density estimates of age, disease duration, PD onset age, and UPDRS-III off-state score. Corresponding statistics are provided in Table S4.

Supplementary Table S4 | Kruskal-Wallis multi-study cohort comparison (Fig. S6)

Cohort characteristic	Kruskal statistic	Kruskal p-value	Post-hoc differences
**Sex**	9·337	0·096	
**Age**	**24·590**	**0·0002**	Eisinger 2020 > Pardo-Valencia 2024 (p=0·008)<br>Eisinger 2020 > Wilkins 2023 (p=0·025)<br>Martin 2018 > Wilkins 2023 (p=0·042)<br>Martin 2018 > Pardo-Valencia 2024 (p=0·014)
**Disease duration**	8·439	0·077	Missing data: Eisinger 2020
**PD onset age**	8·297	0·081	Missing data: Eisinger 2020
**UPDRS-III (off-state)**	**15·742**	**0·003**	Wilkins 2023 (post) > Lofredi 2023 (pre & post) (p=0·008)<br><br>Missing data: Martin 2018

Multi-study cohort comparison including this study's pooled cohort, Lofredi 2023, Wilkins 2023, Pardo-Valencia 2024, Eisinger 2020, and Martin 2018. Bonferroni-corrected alpha for Kruskal-Wallis test: $p = 0 \cdot 01$. Dunn's post hoc p-values are Bonferroni-adjusted. ">" denotes directionality of significant post-hoc differences. "pre"/"post" indicates whether UPDRS was assessed before or after DBS surgery. Note that the pooled cohort from this study did not differ significantly from any other published cohort.

Confounding effects of demographic variables on correlations

We used linear mixed-effects models to reevaluate the correlations between STN activity and motor symptom severity reported in Fig. 4. Dataset identity ($n = 5$) was included as a random intercept, with sex, age, and disease duration as fixed covariates. The analysis included 112 patients; cases with missing demographic data were excluded.

In the relative model (Fig. 4a), relative low beta remained significantly associated with motor symptom severity ($p = 0 \cdot 002$), whereas sex ($p = 0 \cdot 290$), age ($p = 0 \cdot 152$), and disease duration ($p = 0 \cdot 125$) were not significant:

```
          Mixed Linear Model Regression Results
===========================================================
Model:              MixedLM  Dependent Variable:  UPDRS_III
No. Observations:   112      Method:              REML
No. Groups:         5        Scale:               136.4009
Min. group size:    10       Log-Likelihood:      -431.1682
Max. group size:    41       Converged:           Yes
Mean group size:    22.4
-----------------------------------------------------------
                  Coef.  Std.Err.   z   P>|z| [0.025 0.975]
-----------------------------------------------------------
Intercept         12.217    9.548 1.280 0.201 -6.496 30.930
Sex[T.male]        2.705    2.554 1.059 0.290 -2.301  7.710
Relative_Low_Beta 19.848    6.557 3.027 0.002  6.996 32.701
Age                0.202    0.141 1.433 0.152 -0.074  0.479
Disease_Duration   0.396    0.258 1.534 0.125 -0.110  0.902
project Var       35.097    2.833
===========================================================
```

In the absolute model (Fig. 4b), absolute total theta ($p = 7e^{-5}$), low beta ($p = 4e^{-4}$), and low gamma ($p = 0 \cdot 026$) were significantly associated with motor symptom severity. Sex ($p = 0 \cdot 438$), age ($p = 0 \cdot 471$), and disease duration ($p = 0 \cdot 097$) were not significant:

```
            Mixed Linear Model Regression Results
===============================================================
Model:                MixedLM   Dependent Variable:   UPDRS_III
No. Observations:     112       Method:               REML
No. Groups:           5         Scale:                132.4237
Min. group size:      10        Log-Likelihood:       -423.1541
Max. group size:      41        Converged:            Yes
Mean group size:      22.4
---------------------------------------------------------------
                    Coef.  Std.Err.   z     P>|z|  [0.025 0.975]
---------------------------------------------------------------
Intercept           16.368    9.315  1.757 0.079  -1.889 34.625
Sex[T.male]          1.954    2.517  0.776 0.438  -2.980  6.888
Absolute_Theta     -12.565    3.170 -3.963 0.000 -18.778 -6.351
Absolute_Low_Beta   12.620    3.570  3.535 0.000   5.624 19.616
Absolute_Low_Gamma  -9.104    4.085 -2.229 0.026 -17.110 -1.098
Age                  0.102    0.141  0.720 0.471  -0.175  0.379
Disease_Duration     0.416    0.250  1.660 0.097  -0.075  0.906
project Var          6.763    0.840
===============================================================
```

In the periodic model (Fig. 4c), the intercept ($p = 0 \cdot 010$), aperiodic offset ($p = 0 \cdot 016$), periodic low beta ($p = 0 \cdot 002$), and periodic low gamma ($p = 0 \cdot 011$) were significantly associated with motor symptom severity. Sex ($p = 0 \cdot 344$), age ($p = 0 \cdot 363$), and disease duration ($p = 0 \cdot 089$) were not significant:

```
           Mixed Linear Model Regression Results
===============================================================
Model:                  MixedLM   Dependent Variable:   UPDRS_III
No. Observations:       112       Method:               REML
No. Groups:             5         Scale:                132.5274
Min. group size:        10        Log-Likelihood:       -422.1281
Max. group size:        41        Converged:            Yes
Mean group size:        22.4
---------------------------------------------------------------
                    Coef.  Std.Err.   z    P>|z|  [0.025 0.975]
---------------------------------------------------------------
Intercept           23.822    9.265  2.571 0.010   5.663 41.982
Sex[T.male]          2.388    2.526  0.946 0.344  -2.562  7.339
Aperiodic_Offset    -5.197    2.162 -2.404 0.016  -9.435 -0.960
Periodic_Low_Beta   13.675    4.381  3.121 0.002   5.088 22.263
Periodic_Low_Gamma -33.846   13.271 -2.550 0.011 -59.856 -7.835
Age                  0.126    0.138  0.910 0.363  -0.145  0.397
Disease_Duration     0.429    0.252  1.700 0.089  -0.066  0.924
project Var          9.406    1.155
===============================================================
```

The initial analysis focused on sex, age, and disease duration because these variables were available for nearly all patients ($n = 112$). PD onset age was excluded due to collinearity with age and disease duration, which prevents model convergence. Days from surgery to recording was also excluded because it reduced the available sample size to $n = 103$.

To assess these covariates, we ran separate models including PD onset age and days from surgery to recording. For all three models (relative, absolute, periodic), neither PD onset age ($p > 0 \cdot 70$) nor days after surgery ($p > 0 \cdot 62$) were significantly related to motor severity, and the band-power predictors remained significant.

Relative model:

```
          Mixed Linear Model Regression Results
============================================================
Model:                 MixedLM  Dependent Variable:  UPDRS_III
No. Observations:      103      Method:              REML
No. Groups:            5        Scale:               144.2367
Min. group size:       10       Log-Likelihood:      -400.0661
Max. group size:       32       Converged:           Yes
Mean group size:       20.6
------------------------------------------------------------
                   Coef.  Std.Err.   z   P>|z| [0.025 0.975]
------------------------------------------------------------
Intercept          25.868    7.868 3.288 0.001 10.447 41.290
Relative_Low_Beta  22.183    7.044 3.149 0.002  8.376 35.990
PD_onset_age        0.050    0.132 0.378 0.705 -0.209  0.309
Days_after_Surgery  0.490    1.186 0.413 0.680 -1.835  2.815
project Var        42.584    3.304
============================================================
```

Absolute model:

```
            Mixed Linear Model Regression Results
================================================================
Model:                MixedLM   Dependent Variable:   UPDRS_III
No. Observations:     103       Method:               REML
No. Groups:           5         Scale:                132.2810
Min. group size:      10        Log-Likelihood:       -389.4566
Max. group size:      32        Converged:            Yes
Mean group size:      20.6
----------------------------------------------------------------
                    Coef.  Std.Err.   z    P>|z|  [0.025 0.975]
----------------------------------------------------------------
Intercept           27.690    7.458  3.713 0.000  13.074 42.307
Absolute_Theta     -14.735    3.335 -4.419 0.000 -21.270 -8.199
Absolute_Low_Beta   15.549    3.673  4.233 0.000   8.349 22.749
Absolute_Low_Gamma -12.107    4.303 -2.813 0.005 -20.541 -3.672
PD_onset_age        -0.049    0.128 -0.381 0.703  -0.299  0.202
Days_after_Surgery   0.067    0.964  0.069 0.945  -1.823  1.956
project Var          8.044    1.065
================================================================
```

Periodic model:

```
            Mixed Linear Model Regression Results
=================================================================
Model:               MixedLM    Dependent Variable:    UPDRS_III
No. Observations:    103        Method:                REML
No. Groups:          5          Scale:                 128.0310
Min. group size:     10         Log-Likelihood:        -386.9853
Max. group size:     32         Converged:             Yes
Mean group size:     20.6
-----------------------------------------------------------------
                    Coef.  Std.Err.   z    P>|z|  [0.025  0.975]
-----------------------------------------------------------------
Intercept           37.519    7.122  5.268 0.000  23.560  51.478
Aperiodic_Offset    -6.525    2.104 -3.102 0.002 -10.648  -2.402
Periodic_Low_Beta   18.169    4.514  4.025 0.000   9.322  27.017
Periodic_Low_Gamma -44.525   14.485 -3.074 0.002 -72.915 -16.135
PD_onset_age        -0.025    0.123 -0.202 0.840  -0.265   0.216
Days_after_Surgery   0.503    1.016  0.495 0.621  -1.489   2.495
project Var         13.898    1.454
=================================================================
```

In summary, none of the tested covariates (sex, age, disease duration, PD onset age, days between surgery and recording) confounded the reported correlations. All significant multiple linear regression parameters in Fig. 4 remained significant when using linear mixed-effects models.

Part 2: Spectral framework comparison

Theta, low beta, and low gamma oscillations explain unique variance of motor symptom severity

Correlation analysis (Fig. 3h) indicated a prokinetic role of theta and low gamma activity, and an antikinetic role of low beta oscillations. To test whether reductions in beta power could explain the theta effect, we fit a linear mixed-effects model including theta, low beta, and low gamma band power as predictors of UPDRS-III scores.

Both periodic theta ($p = 0 \cdot 044$) and periodic low beta ($p = 0 \cdot 002$) remained significant, indicating that each contributes unique variance to motor symptom severity. Periodic low gamma also remained significant ($p = 0 \cdot 005$). These results suggest that the observed theta association is not merely a byproduct of beta-band changes, but rather reflects an additional relationship with motor symptoms.

```
             Mixed Linear Model Regression Results
================================================================
Model:                  MixedLM     Dependent Variable:    UPDRS_III
No. Observations:       115         Method:                REML
No. Groups:             5           Scale:                 130.3401
Min. group size:        10          Log-Likelihood:        -434.8646
Max. group size:        42          Converged:             Yes
Mean group size:        23.0
----------------------------------------------------------------
                       Coef.  Std.Err.   z    P>|z|  [0.025  0.975]
----------------------------------------------------------------
Intercept              37.866    3.285 11.528 0.000  31.429  44.304
Periodic_Theta        -15.851    7.857 -2.017 0.044 -31.251  -0.451
Periodic_Low_Beta      13.600    4.290  3.170 0.002   5.192  22.007
Periodic_Low_Gamma    -37.007   13.161 -2.812 0.005 -62.802 -11.211
project Var            29.460    2.412
================================================================
```

Part 3: Within-patient correlations

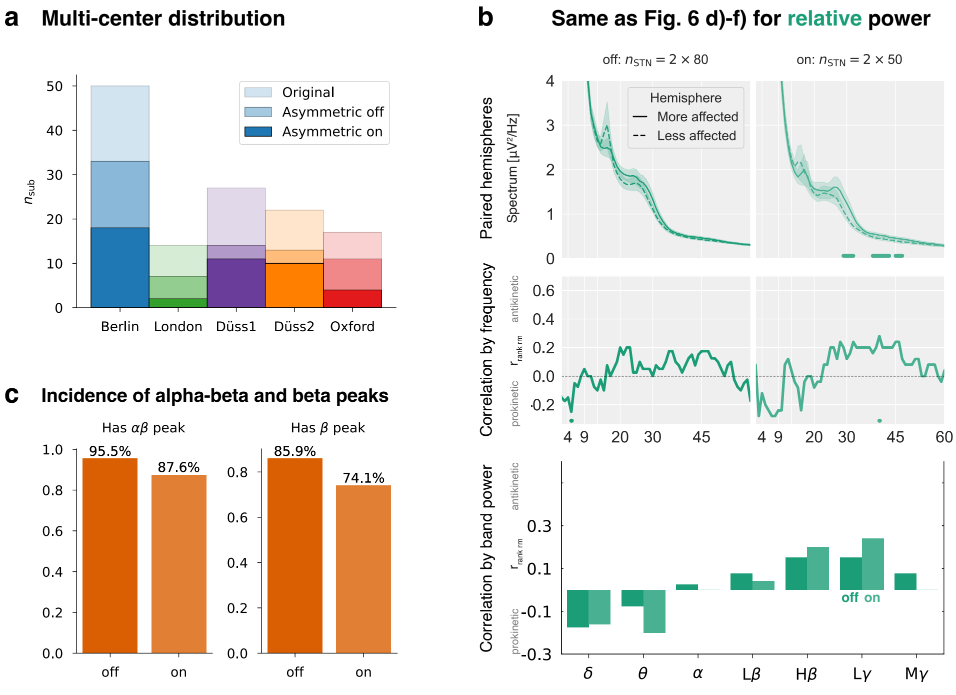

Supplementary Fig. S7 | Supplementary information for within-patient investigation in Figs. 6-7. a, Within-patient analysis inclusion by dataset and Levodopa condition. **b**, Same as **Fig. 6d- f** for relative power. **c**, *specparam*'s probability of fitting a peak in a given frequency band. Same as **Fig. 7b** for alpha-beta and beta. Only when considering the full alpha-beta range (8–35 Hz) did at least 88% of STNs have a peak, as previously shown.[52–54]

Spatial specificity of spectral biomarkers

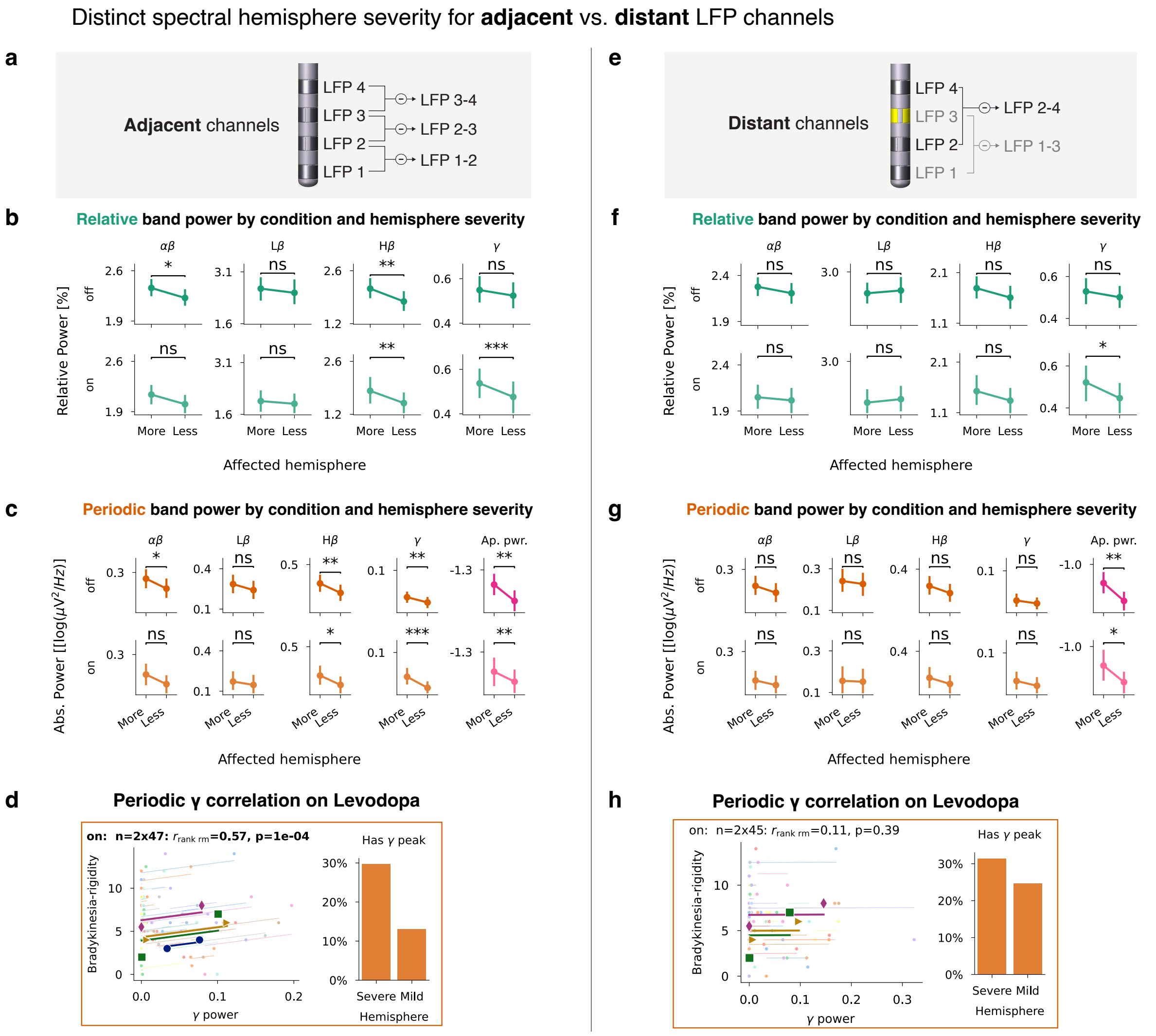

Supplementary Fig. S8 | Distinct spectral hemisphere severity for adjacent vs. distant LFP channels. a, Visualisation of adjacent bipolar LFP montage. **b**, Wilcoxon signed-rank test for relative band power comparing the more and less affected hemispheres in the Levodopa off- and on-state. **c**, Same as **b** for absolute periodic power. **d**, Left: Repeated measures correlation for periodic gamma oscillations in the Levodopa on-state. Four highlighted subjects are shown in **Supplementary Fig. S8d**. Right: *specparam*'s probability of fitting gamma peaks by hemisphere. **e-h**, Same as **a-d** for distant LFP channels recommended for aDBS. Note that alpha-beta and gamma are only elevated for the more affected hemisphere in the Levodopa off- or on-state, respectively, and they are not robust with regard to the spatial specificity of the bipolar montage. In contrast, aperiodic broadband power is elevated in the more affected hemisphere independent of Levodopa medication and channel choice: Adjacent channels off: $p = 0 \cdot 008$, on: $p = 0 \cdot 009$; distant channels off: $p = 0 \cdot 005$, on: $p = 0 \cdot 015$.

DBS electrodes enable LFP sensing, usually via a bipolar reference montage. Some studies focus on adjacent DBS channels (1–2, 2–3, or 3–4, Supplementary Fig. S8a), whereas others use distant channels (1–3 or 2–4, Supplementary Fig. S8e). Adjacent channels better capture local dynamics, whereas distant channels are spatially less specific. When analysing adjacent bipolar channels, we observe a strong within-patient correlation ($r_{\text{rank rm}} = 0 \cdot 57, p = 1e^{-4}$) between bradykinesia-rigidity symptoms and periodic gamma oscillations in the Levodopa on-state (Supplementary Fig. S9b). These STN gamma oscillations are highlighted in yellow in Supplementary Fig. S9d and were reported before.[55,56] However, their local specificity limits their potential as a reliable aDBS biomarker. An optimal aDBS biomarker

should be detectable from distant LFP channels, such as 1–3 or 2–4, which are adjacent to the central stimulation contacts (2 or 3). For distant channels (1–3 or 2–4), only absolute total mid gamma power and aperiodic broadband power correlated significantly with patients in the Levodopa off- and on-state.

Results for **adjacent** bipolar LFP channels 1-2, 2-3, or 3-4

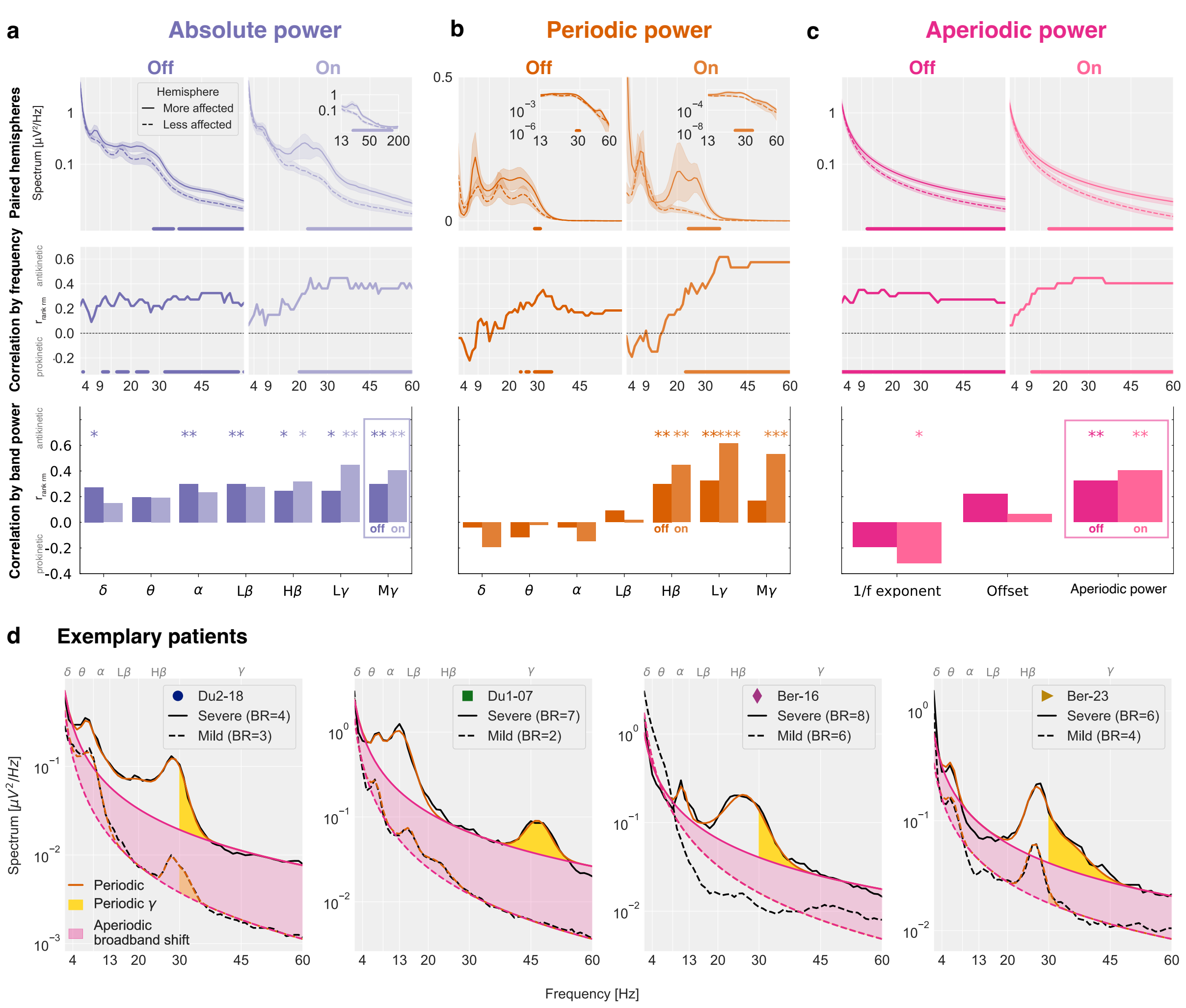

Supplementary Fig. S9 | Periodic gamma oscillations correlate with motor symptom severity within patients on Levodopa. Same as Figs. 6-7 for adjacent instead of distant bipolar LFP channels. **a**, Top: Within-patients paired cluster-based permutation tests for the more and less affected hemisphere. Middle: Repeated measures rank correlation for each frequency bin of the power spectrum and the contralateral bradykinesia-rigidity subscore. Bottom: Repeated measures rank correlation for band powers. **b-c**, Same as **a** for periodic and aperiodic power. **d**, Exemplary patients. The symbols of each patient correspond to the symbols in the repeated measures scatter plots in Supplementary **Fig. S8d** and **S8h**. Gamma oscillations often overlap with high beta oscillations (Du2-18, Ber-16, Ber-23), but they correlate more strongly with symptoms than high beta power. Note that the periodic gamma oscillation in patient Du1-07 is more prominent in the adjacent bipolar montage compared to the distant bipolar montage shown in **Fig. 7**. This suggests that periodic gamma oscillations carry pathological information but are spatially narrowly localised, hindering their utility as aDBS biomarker.

Influence of tremor on within-patient correlations

To address whether mid gamma power also captures tremor severity, we repeated our within-patient analyses using mid gamma power and UPDRS hemibody scores that included bradykinesia, rigidity, and tremor subscores. Compared to bradykinesia-rigidity alone (off: $n = 78$, $r_{rank\ rm} = 0 \cdot 38$, $p = 0 \cdot 0004$; on: $n = 45$, $r_{rank\ rm} = 0 \cdot 38$, $p = 0 \cdot 008$),

hemibody correlations were slightly weaker but remained significant (off: $n = 72$, $r_{rank\ rm} = 0 \cdot 36, p = 0 \cdot 003$; on: $n = 41$, $r_{rank\ rm} = 0 \cdot 32, p = 0 \cdot 036$, Supplementary Fig. S10b). Tremor-only analyses yielded no significant correlations (off: $n = 43$, $r_{rank\ rm} = 0 \cdot 12, p = 0 \cdot 425$; on: $n = 13$, $r_{rank\ rm} = 0 \cdot 38, p = 0 \cdot 157$, Supplementary Fig. S10e). Similar results were obtained for aperiodic broadband power (Supplementary Fig. S10c, f). Because tremor scores were generally low, many patients were excluded due to identical scores on both body sides, which reduced the effective sample size and limited interpretability. These results suggest that mid gamma activity is more strongly related to bradykinesia-rigidity than to tremor in this cohort, although the latter relationship warrants further investigation in larger, more tremor-dominant samples. Notably, beta power, the first FDA-approved aDBS biomarker,[57] also does not correlate with tremor;[28,30,41,43,58–61] yet, it has nevertheless proven clinically useful.

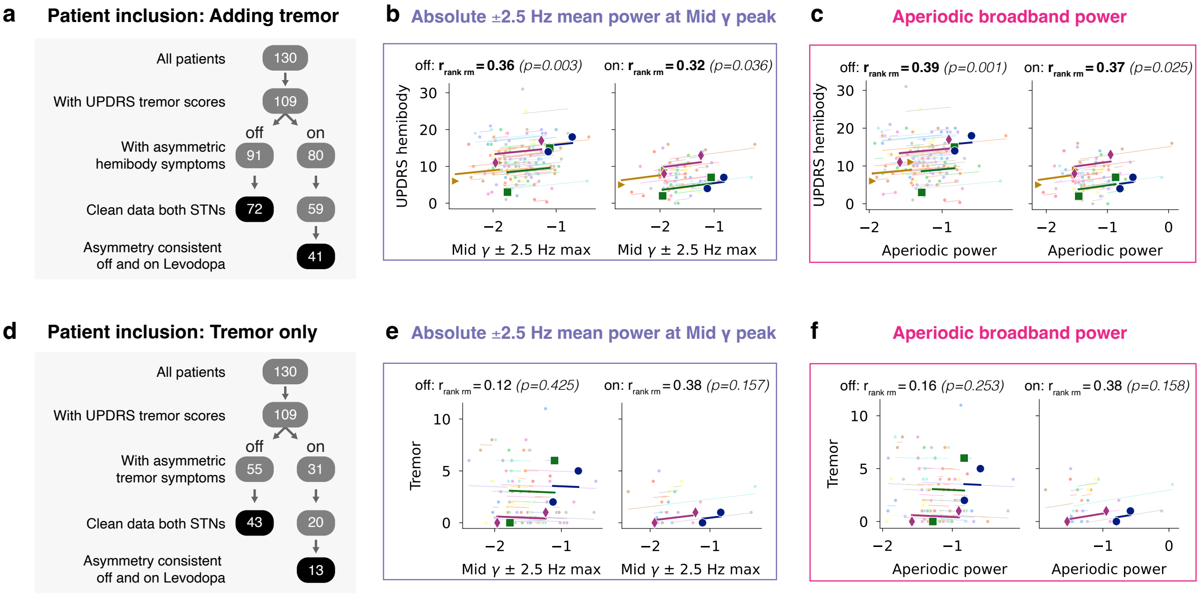

Supplementary Fig. S10 | Effect of including tremor in within-patient correlations between aperiodic broadband power and motor symptom severity. a) Same as Fig. 6b, but using UPDRS hemibody subscores that include bradykinesia, rigidity, and tremor. Tremor data were unavailable for the Oxford dataset, slightly reducing the sample size. b) Same as Fig. 7a, but with tremor included in the hemibody subscores. c) Same as Fig. 7c, but with tremor included. d) Same as a) for tremor subscores only. Low tremor scores increase the likelihood of equal scores for both body sides, substantially reducing the available sample size. (e-f) Same as (b-c) for tremor only.

Relationship between mid gamma power and aperiodic parameters

We used simulations to examine how mid gamma power reflects aperiodic parameters. Varying the offset (while fixing the 1/f exponent at 1) showed a correlation of $r = 1$ between offset and mid gamma power (Supplementary Fig. S11a–b). Mid gamma power also correlated with aperiodic broadband power at $r = 1$ (Supplementary Fig. S11c). Varying the 1/f exponent (offset fixed at 1) produced an inverse correlation between exponent and mid gamma power at $r = -1$ (Supplementary Fig. S11d–e), and a positive correlation with aperiodic broadband power at $r = 1$ (Supplementary Fig. S11f).

We then tested these relationships in our empirical data. Mid gamma power correlated significantly with both aperiodic offset ($r = 0 \cdot 24, p = 0 \cdot 008$, Supplementary Fig. S11g) and the 1/f exponent ($r = -0 \cdot 24, p = 0 \cdot 007$, Supplementary Fig. S11h). The strongest

correlation, however, was with aperiodic broadband power ($r = 0 \cdot 93, p = 0 \cdot 0001$; Supplementary Fig. S11i).

The empirical results show that mid gamma power closely tracks aperiodic broadband activity, more so than offset or slope separately. Whereas estimating aperiodic broadband power requires computationally demanding spectral parameterisation, mid gamma power can be readily extracted in real time from existing sensing-enabled DBS systems (e.g., Medtronic Percept™ PC), supporting its utility as a practical aDBS biomarker.

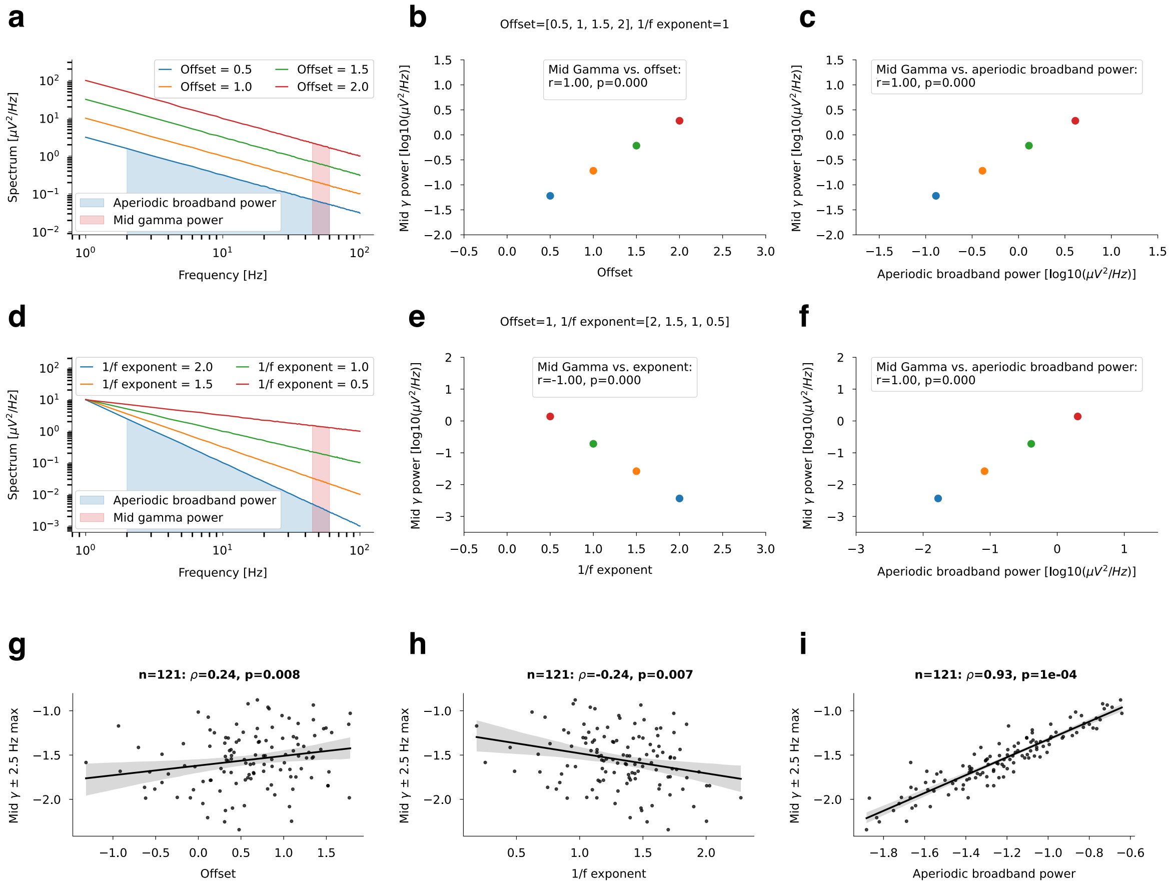

Supplementary Fig. S11 | Relationship between mid gamma power and aperiodic parameters. Simulations (a–f) and empirical results (g–i) illustrating how mid gamma power relates to the aperiodic offset, the 1/f exponent, and aperiodic broadband power. a) Simulated spectra with varying aperiodic offsets (0·5 to 2, exponent fixed at 1). Aperiodic broadband power (2–60 Hz) is shown in blue for an offset of 0·5, mid gamma power in red for an offset of 2. b) Colored dots correspond to spectra in a), correlation between offset and mid gamma power is shown. c) Relationship between mid gamma power and aperiodic broadband power. (d-f) Same as (a-c) with varying 1/f exponents (0·5 to 2, offset fixed at 1). g) Empirical correlation between mid gamma power and aperiodic offset (off-state). h) Same as g) for 1/f exponent. i) Same as g) for aperiodic broadband power.

Age-related effects on subthalamic spectral features

To test whether age influences subthalamic spectral features, we first correlated age with absolute power at each frequency bin. Significant negative correlations emerged for ~23–100 Hz (Supplementary Fig. S12a). Among the aperiodic parameters, neither the offset nor the 1/f exponent correlated with age, but aperiodic broadband power showed a significant negative association ($r = -0 \cdot 24, p = 0 \cdot 009$; Supplementary Fig. S12b–d).

We then repeated the within-patient correlation between aperiodic broadband power and motor symptoms (Fig. 7c) separately for younger and older patients (median split). In younger patients, correlations were significant in the off-state ($r_{rank\ rm} = 0 \cdot 35, p = 0 \cdot 031$) but not in the on-state ($r_{rank\ rm} = 0 \cdot 36, p = 0 \cdot 105$). In older patients, correlations remained significant in both medication states (off: $r_{rank\ rm} = 0 \cdot 46, p = 0 \cdot 006$; on: $r_{rank\ rm} = 0 \cdot 48, p = 0 \cdot 014$; Supplementary Fig. S12e–f). The absence of significance in the younger on-state split likely reflects reduced statistical power, as the sample was small ($n = 22$) yet still yielded a moderate effect size ($|r| > 0 \cdot 3$).[62]

For frequencies <100 Hz, our findings align with Martin et al.,[43] who reported a similar frequency-dependent pattern in STN power, with low frequencies uncorrelated with age, and intermediate frequencies being negatively correlated (see their Fig. 2c). While they observed significant positive correlations for frequencies >150 Hz, we found negative non-significant correlations in this range. However, their small cohort ($n = 13$) limits interpretability.

In the cortex, multiple studies have reported that age reduces the 1/f exponent and aperiodic offset,[22,63–67;] however, recent work has suggested that these effects may partly reflect ECG artefacts.[68] To our knowledge, no prior studies have examined age effects on STN aperiodic parameters. Interestingly, while neither the STN offset nor exponent correlated with age, aperiodic broadband power did—showing that it correlates positively with motor symptoms within patients but negatively with age across patients.

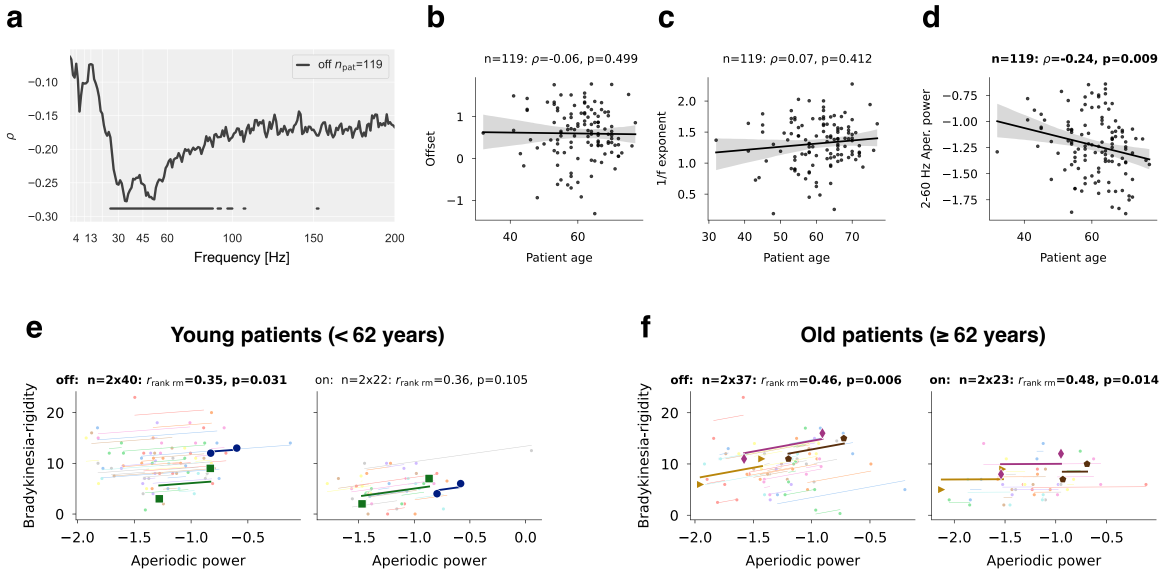

Supplementary Fig. S12 | Impact of age on spectral power, aperiodic parameters, and within-patient correlations. a) Frequency-wise correlation between age and absolute spectral power. Horizontal lines indicate frequencies with uncorrected p-values $< 0 \cdot 05$. b-d) Scatter plots showing correlations between patient age and aperiodic offset, 1/f exponent, and aperiodic broadband power. e-f) Within-patient correlations between aperiodic broadband power and motor symptoms (as in Fig. 7c), shown separately for e) younger (< 62 years) and f) older patients (≥ 62 years).

References

1 Köhler RM, Binns TS, Merk T, Zhu G, Yin Z, Zhao B, et al. Dopamine and deep brain stimulation accelerate the neural dynamics of volitional action in Parkinson's disease. Brain 2024;147:3358–69.

2 Busch JL, Kaplan J, Habets JGV, Feldmann LK, Roediger J, Köhler RM, et al. Single threshold adaptive deep brain stimulation in Parkinson's disease depends on parameter selection, movement state and controllability of subthalamic beta activity. Brain Stimul 2024;17:125–33.

3 Binns TS, Köhler RM, Vanhoecke J, Chikermane M, Gerster M, Merk T, et al. Shared pathway-specific network mechanisms of dopamine and deep brain stimulation for the treatment of Parkinson's disease. Nat Commun 2025;16:3587.

4 Litvak V, Jha A, Eusebio A, Oostenveld R, Foltynie T, Limousin P, et al. Resting oscillatory cortico-subthalamic connectivity in patients with Parkinson's disease. Brain 2011;134:359–74.

5 Sharma A, Vidaurre D, Vesper J, Schnitzler A, Florin E. Differential dopaminergic modulation of spontaneous cortico-subthalamic activity in Parkinson's disease. Elife 2021;10:e66057.

6 Sure M, Vesper J, Schnitzler A, Florin E. Dopaminergic modulation of spectral and spatial characteristics of parkinsonian subthalamic nucleus beta bursts. Front Neurosci 2021;15:724334.

7 Sure M, Vesper J, Schnitzler A, Florin E. Cortical network formation based on subthalamic beta bursts in Parkinson's disease. Neuroimage 2022;263:119619.

8 Hirschmann J, Steina A, Vesper J, Florin E, Schnitzler A. Neuronal oscillations predict deep brain stimulation outcome in Parkinson's disease. Brain Stimul 2022;15:792–802.

9 Sure M, Mertiens S, Vesper J, Schnitzler A, Florin E. Alterations of resting-state networks of Parkinson's disease patients after subthalamic DBS surgery. NeuroImage Clin 2023;37:103317.

10 Rassoulou F, Steina A, Hartmann CJ, Vesper J, Butz M, Schnitzler A, et al. Exploring the electrophysiology of Parkinson's disease with magnetoencephalography and deep brain recordings. Sci Data 2024;11:889.

11 Hirschmann J, Özkurt TE, Butz M, Homburger M, Elben S, Hartmann CJ, et al. Differential modulation of STN-cortical and cortico-muscular coherence by movement and levodopa in Parkinson's disease. Neuroimage 2013;68:203–13.

12 Hirschmann J, Özkurt TE, Butz M, Homburger M, Elben S, Hartmann CJ, et al. Distinct oscillatory STN-cortical loops revealed by simultaneous MEG and local field potential recordings in patients with Parkinson's disease. Neuroimage 2011;55:1159–68.

13 Wiest C, Torrecillos F, Pogosyan A, Bange M, Muthuraman M, Groppa S, et al. The aperiodic exponent of subthalamic field potentials reflects excitation/inhibition balance in Parkinsonism. Elife 2023;12:e82467.

14 Wiest C, Tinkhauser G, Pogosyan A, He S, Baig F, Morgante F, et al. Subthalamic deep brain stimulation induces finely-tuned gamma oscillations in the absence of levodopa. Neurobiol Dis 2021;152:105287.

15 Wiest C, Tinkhauser G, Pogosyan A, Bange M, Muthuraman M, Groppa S, et al. Local field potential activity dynamics in response to deep brain stimulation of the subthalamic nucleus in Parkinson's disease. Neurobiol Dis 2020;143:105019.

16 Harris CR, Millman KJ, van der Walt SJ, Gommers R, Virtanen P, Cournapeau D, et al. Array programming with NumPy. Nature 2020;585:357–62.

17 Virtanen P, Gommers R, Oliphant TE, Haberland M, Reddy T, Cournapeau D, et al. SciPy 1.0: fundamental algorithms for scientific computing in Python. Nat Methods 2020;17:261–72.

18 Gramfort A, Luessi M, Larson E, Engemann DA, Strohmeier D, Brodbeck C, et al. MEG and EEG data analysis with MNE-Python. Front Neurosci 2013;7:267.

19 Appelhoff S, Sanderson M, Brooks TL, van Vliet M, Quentin R, Holdgraf C, et al. MNE-BIDS: Organizing electrophysiological data into the BIDS format and facilitating their analysis. J Open Source Softw 2019;4:1896.

20 The pandas development team. pandas-dev/pandas: Pandas. Zenodo; 2020. https://doi.org/10.5281/ZENODO.13819579.

21 McKinney W. Data Structures for Statistical Computing in Python. Proceedings of the Python in Science Conference, SciPy; 2010, p. 56–61.

22 Donoghue T, Haller M, Peterson EJ, Varma P, Sebastian P, Gao R, et al. Parameterizing neural power spectra into periodic and aperiodic components. Nat Neurosci 2020;23:1655–65.

23 Waskom M. seaborn: statistical data visualization. J Open Source Softw 2021;6:3021.

24 Vallat R. Pingouin: statistics in Python. J Open Source Softw 2018;3:1026.

25 Seabold S, Perktold J. Statsmodels: Econometric and statistical modeling with python. Proceedings of the Python in Science Conference, vol. 2010, SciPy; 2010, p. 92–6.

26 Penny WD, Friston KJ, Ashburner JT, Kiebel SJ, Nichols TE, editors. Statistical parametric mapping: The

analysis of functional brain images. San Diego, CA: Academic Press; 2006.
27 Neudorfer C, Butenko K, Oxenford S, Rajamani N, Achtzehn J, Goede L, et al. Lead-DBS v3.0: Mapping deep brain stimulation effects to local anatomy and global networks. Neuroimage 2023;268:119862.
28 Kühn AA, Kupsch A, Schneider G-H, Brown P. Reduction in subthalamic 8-35 Hz oscillatory activity correlates with clinical improvement in Parkinson's disease: STN activity and motor improvement. Eur J Neurosci 2006;23:1956–60.
29 Ray NJ, Jenkinson N, Wang S, Holland P, Brittain JS, Joint C, et al. Local field potential beta activity in the subthalamic nucleus of patients with Parkinson's disease is associated with improvements in bradykinesia after dopamine and deep brain stimulation. Exp Neurol 2008;213:108–13.
30 Kühn AA, Tsui A, Aziz T, Ray N, Brücke C, Kupsch A, et al. Pathological synchronisation in the subthalamic nucleus of patients with Parkinson's disease relates to both bradykinesia and rigidity. Exp Neurol 2009;215:380–7.
31 Chen CC, Hsu YT, Chan HL, Chiou SM, Tu PH, Lee ST, et al. Complexity of subthalamic 13-35 Hz oscillatory activity directly correlates with clinical impairment in patients with Parkinson's disease. Exp Neurol 2010;224:234–40.
32 López-Azcárate J, Tainta M, Rodríguez-Oroz MC, Valencia M, González R, Guridi J, et al. Coupling between beta and high-frequency activity in the human subthalamic nucleus may be a pathophysiological mechanism in Parkinson's disease. J Neurosci 2010;30:6667–77.
33 Özkurt TE, Butz M, Homburger M, Elben S, Vesper J, Wojtecki L, et al. High frequency oscillations in the subthalamic nucleus: a neurophysiological marker of the motor state in Parkinson's disease. Exp Neurol 2011;229:324–31.
34 Hohlefeld FU, Huebl J, Huchzermeyer C, Schneider G-H, Schönecker T, Kühn AA, et al. Long-range temporal correlations in the subthalamic nucleus of patients with Parkinson's disease. Eur J Neurosci 2012;36:2812–21.
35 Little S, Pogosyan A, Kuhn AA, Brown P. β band stability over time correlates with Parkinsonian rigidity and bradykinesia. Exp Neurol 2012;236:383–8.
36 Trager MH, Koop MM, Velisar A, Blumenfeld Z, Nikolau JS, Quinn EJ, et al. Subthalamic beta oscillations are attenuated after withdrawal of chronic high frequency neurostimulation in Parkinson's disease. Neurobiol Dis 2016;96:22–30.
37 Neumann W-J, Degen K, Schneider G-H, Brücke C, Huebl J, Brown P, et al. Subthalamic synchronized oscillatory activity correlates with motor impairment in patients with Parkinson's disease. Mov Disord 2016;31:1748–51.
38 West T, Farmer S, Berthouze L, Jha A, Beudel M, Foltynie T, et al. The parkinsonian subthalamic network: Measures of power, linear, and non-linear synchronization and their relationship to L-DOPA treatment and OFF state motor severity. Front Hum Neurosci 2016;10:517.
39 Beudel M, Oswal A, Jha A, Foltynie T, Zrinzo L, Hariz M, et al. Oscillatory beta power correlates with akinesia-rigidity in the parkinsonian subthalamic nucleus. Mov Disord 2017;32:174–5.
40 Neumann W-J, Staub-Bartelt F, Horn A, Schanda J, Schneider G-H, Brown P, et al. Long term correlation of subthalamic beta band activity with motor impairment in patients with Parkinson's disease. Clin Neurophysiol 2017;128:2286–91.
41 Neumann W-J, Kühn AA. Subthalamic beta power-Unified Parkinson's disease rating scale III correlations require akinetic symptoms. Mov Disord 2017;32:175–6.
42 van Wijk BCM, Pogosyan A, Hariz MI, Akram H, Foltynie T, Limousin P, et al. Localization of beta and high-frequency oscillations within the subthalamic nucleus region. NeuroImage Clin 2017;16:175–83.
43 Martin S, Iturrate I, Chavarriaga R, Leeb R, Sobolewski A, Li AM, et al. Differential contributions of subthalamic beta rhythms and 1/f broadband activity to motor symptoms in Parkinson's disease. NPJ Parkinsons Dis 2018;4:32.
44 Ozturk M, Abosch A, Francis D, Wu J, Jimenez-Shahed J, Ince NF. Distinct subthalamic coupling in the ON state describes motor performance in Parkinson's disease. Mov Disord 2020;35:91–100.
45 Eisinger RS, Cagle JN, Opri E, Alcantara J, Cernera S, Foote KD, et al. Parkinsonian beta dynamics during rest and movement in the dorsal pallidum and subthalamic nucleus. J Neurosci 2020;40:2859–67.
46 Averna A, Marceglia S, Arlotti M, Locatelli M, Rampini P, Priori A, et al. Influence of inter-electrode distance on subthalamic nucleus local field potential recordings in Parkinson's disease. Clin Neurophysiol 2022;133:29–38.
47 Lofredi R, Okudzhava L, Irmen F, Brücke C, Huebl J, Krauss JK, et al. Subthalamic beta bursts correlate with dopamine-dependent motor symptoms in 106 Parkinson's patients. NPJ Parkinsons Dis 2023;9:2.
48 Pardo-Valencia J, Fernández-García C, Alonso-Frech F, Foffani G. Oscillatory vs. non-oscillatory subthalamic beta activity in Parkinson's disease. J Physiol 2024;602:373–95.
49 Wilkins KB, Kehnemouyi YM, Petrucci MN, Anderson RW, Parker JE, Trager MH, et al. Bradykinesia and its progression are related to interhemispheric beta coherence. Ann Neurol 2023;93:1029–39.

50 Mestre TA, Lang AE, Okun MS. Factors influencing the outcome of deep brain stimulation: Placebo, nocebo, lessebo, and lesion effects: Factors Influencing Deep Brain Stimulation Outcome. Mov Disord 2016;31:290–6.
51 Mann JM, Foote KD, Garvan CW, Fernandez HH, Jacobson CE 4th, Rodriguez RL, et al. Brain penetration effects of microelectrodes and DBS leads in STN or GPi. J Neurol Neurosurg Psychiatry 2009;80:794–7.
52 Darcy N, Lofredi R, Al-Fatly B, Neumann W-J, Hübl J, Brücke C, et al. Spectral and spatial distribution of subthalamic beta peak activity in Parkinson's disease patients. Exp Neurol 2022;356:114150.
53 Shreve LA, Velisar A, Malekmohammadi M, Koop MM, Trager M, Quinn EJ, et al. Subthalamic oscillations and phase amplitude coupling are greater in the more affected hemisphere in Parkinson's disease. Clin Neurophysiol 2017;128:128–37.
54 Behnke JK, Peach RL, Habets JGV, Busch JL, Kaplan J, Roediger J, et al. Long-term stability of spatial distribution and peak dynamics of subthalamic beta power in Parkinson's disease patients. Mov Disord 2025;40:1070–84.
55 Olaru M, Cernera S, Hahn A, Wozny TA, Anso J, de Hemptinne C, et al. Motor network gamma oscillations in chronic home recordings predict dyskinesia in Parkinson's disease. Brain 2024;147:2038–52.
56 Guan L, Yu H, Chen Y, Gong C, Hao H, Guo Y, et al. Subthalamic γ oscillation underlying rapid eye movement sleep abnormality in parkinsonian patients. Mov Disord 2025;40:456–67.
57 Stanslaski S, Summers RLS, Tonder L, Tan Y, Case M, Raike RS, et al. Sensing data and methodology from the Adaptive DBS Algorithm for Personalized Therapy in Parkinson's Disease (ADAPT-PD) clinical trial. NPJ Parkinsons Dis 2024;10:174.
58 Asch N, Herschman Y, Maoz R, Auerbach-Asch CR, Valsky D, Abu-Snineh M, et al. Independently together: subthalamic theta and beta opposite roles in predicting Parkinson's tremor. Brain Commun 2020;2:fcaa074.
59 Sumarac S, Youn J, Fearon C, Zivkovic L, Keerthi P, Flouty O, et al. Clinico-physiological correlates of Parkinson's disease from multi-resolution basal ganglia recordings. NPJ Parkinsons Dis 2024;10:175.
60 Lofredi R, Tan H, Neumann W-J, Yeh C-H, Schneider G-H, Kühn AA, et al. Beta bursts during continuous movements accompany the velocity decrement in Parkinson's disease patients. Neurobiol Dis 2019;127:462–71.
61 Telkes I, Viswanathan A, Jimenez-Shahed J, Abosch A, Ozturk M, Gupte A, et al. Local field potentials of subthalamic nucleus contain electrophysiological footprints of motor subtypes of Parkinson's disease. Proc Natl Acad Sci U S A 2018;115:E8567–76.
62 Loth E, Ahmad J, Chatham C, López B, Carter B, Crawley D, et al. The meaning of significant mean group differences for biomarker discovery. PLoS Comput Biol 2021;17:e1009477.
63 Tröndle M, Popov T, Pedroni A, Pfeiffer C, Barańczuk-Turska Z, Langer N. Decomposing age effects in EEG alpha power. Cortex 2023;161:116–44.
64 Merkin A, Sghirripa S, Graetz L, Smith AE, Hordacre B, Harris R, et al. Do age-related differences in aperiodic neural activity explain differences in resting EEG alpha? Neurobiol Aging 2023;121:78–87.
65 Thuwal K, Banerjee A, Roy D. MEG Oscillatory and Aperiodic neural dynamics contribute to different cognitive aspects of short-term memory decline through lifespan. BioRxiv 2021:2021.03.02.433594.
66 Cellier D, Riddle J, Petersen I, Hwang K. The development of theta and alpha neural oscillations from ages 3 to 24 years. Dev Cogn Neurosci 2021;50:100969.
67 Voytek B, Kramer MA, Case J, Lepage KQ, Tempesta ZR, Knight RT, et al. Age-related changes in 1/f neural electrophysiological noise. J Neurosci 2015;35:13257–65.
68 Schmidt F, Danböck SK, Trinka E, Klein DP, Demarchi G, Weisz N. Age-related changes in "cortical" 1/f dynamics are linked to cardiac activity. Elife 2025;13:RP100605.
